# Nonlinear relationship between serum 25-hydroxyvitamin D and lipid profile in Chinese adults

**DOI:** 10.3389/fnut.2024.1388017

**Published:** 2024-06-12

**Authors:** Qianqian Wang, Xinlei Miao, Manling Hu, Fei Xu, Guimin Tang, Yangxuan He, Ziping Song, Wan Zhao, Xiangjun Niu, Song Leng

**Affiliations:** ^1^Health Management Center, The Second Hospital of Dalian Medical University, Dalian, Liaoning, China; ^2^School of Public Health, Dalian Medical University, Dalian, Liaoning, China; ^3^Department of Gastroenterology, The Second Hospital of Dalian Medical University, Dalian, Liaoning, China

**Keywords:** 25-hydroxyvitamin D, lipid profile, threshold, non-linear, cholesterol

## Abstract

**Background:**

Previous studies on the liner associations between serum 25-hydroxyvitamin D [25(OH)D] levels and lipid profiles revealed ambiguous findings. The current study therefore tried to elucidate the possible non-linear associations between 25(OH)D and lipid profiles.

**Methods:**

This study involved 8,516 adult participants (aged 18–74 years, males *N* = 3,750, females *N* = 4,766) recruited from the Dalian health management cohort (DHMC). The risk (OR) for specific dyslipidemias was estimated across the serum 25(OH)D levels and the cut-off value for serum 25(OH)D were determined by using logistic regression, restricted cubic spline, and piecewise linear regression methods, adjusted for age, sex, season, and ultraviolet index.

**Results:**

In this study, a high prevalence of 25(OH)D deficiency was observed in the participants (65.05%). The level of 25(OH)D showed the inverse U-shaped correlations with the risks (ORs) of abnormal lipid profile, with inflection points observed at 23.7 ng/ml for hypercholesterolemia, 24.3 ng/ml for hypertriglyceridemia, 18.5 ng/ml for hyper-low-density lipoprotein cholesterolemia, 23.3 ng/ml for hypo-high-density lipoprotein cholesterolemia, 23.3 ng/ml for hyper-non-high-density lipoprotein cholesterol, and 24.3 ng/ml for high remnant cholesterol. The stratified analyses showed that the risk for most dyslipidemias related to deficiency of 25(OH)D was particularly increased among females aged 50–74 (except for hypertriglyceridemia, where the highest risk was among men aged 50–74 years), during winter/spring or under low/middle ultraviolet index environments.

**Conclusions:**

Nonlinear inverse U-shaped associations were observed between 25(OH)D levels and abnormal lipid profile. The risk was particularly increased among females aged 50-74, during winter/spring period or under lower ultraviolet index environments. In vitamin D deficient subjects [25(OH)D <20 ng/ml], a positive association of serum vitamin D levels with the risk for dyslipidemia was observed, which needs a further.

## 1 Introduction

The lipid profile includes cholesterol and other lipids present in the bloodstream, such as total cholesterol (TC), triglycerides (TG), high-density lipoprotein cholesterol (HDL-C), low-density lipoprotein cholesterol (LDL-C), and remnant cholesterol (RC) (1–5). Abnormal lipid profile is a characteristic of dyslipidemia and significantly increase the susceptibility to cardiovascular disease (6, 7). Previous studies have consistently shown that elevated plasma levels of low-density lipoprotein particles, triglyceride-rich lipoproteins, and cholesterol-rich remnants significantly heighten the prevalence of atherosclerotic cardiovascular disease (8, 9).

25-hydroxyvitamin D [25(OH)D], a steroid hormone, plays a crucial role in regulating mineral (Ca and P) metabolism in the body and is essential not only for bones but also for other tissues (10). Deficiency of 25(OH)D is not only associated with musculoskeletal disorders, hypertension, and diabetes, but also has gained attention in relation to atherosclerotic dyslipidemia (11–13). Previous research has indicated that a higher serum level of 25(OH)D significantly reduces the likelihood of abnormal lipid profile (14, 15). The prevalence of its deficiency has raised interests among individuals, ranging from 30% to 70% (16–18). Studies have shown that deficiencies in 25(OH)D can be compensated for by exposing the skin to ultraviolet radiation from sunlight and maintaining a balanced diet (19). The concentration of 25(OH)D that can cause 25(OH)D poisoning is 224 ng/ml, while for healthy individuals, the concentration of 25(OH)D should not exceed 150 ng/ml (20, 21). However, the optimal threshold value of 25(OH)D for lipid profile remains uncertain, necessitating further research to establish.

The purpose of this study is to examine the association of serum 25(OH)D levels with abnormal lipid profile among adult Chinese population in Dalian Health Management Cohort (DHMC) and to determine the optimal 25(OH)D concentration associated with the improved lipid status of this population.

## 2 Methods

### 2.1 Study population

This study used the data from the DHMC (ChiCTR2300073363), which was established in 2014 at the Second Hospital of Dalian Medical University. The DHMC encompasses the entire population of Dalian, China. Dalian City is situated between 38°43′-40°12′ north latitude and 120°58′-123°31′ east longitude. The average temperature in winter plunges below −0.5 degrees Celsius.

The study was approved by the Ethics Committee of the Second Hospital of Dalian Medical University (Grant number: 2022064). For this cross-sectional study, 16,409 participants aged 18–74 years were recruited to test 25(OH)D from 2020 to 2023. The study began only after all participants had provided written consent. All the participants underwent a comprehensive examination that included physical, laboratory, imaging tests, and questionnaires.

Participants were excluded based on the following criteria: oncologic patients (*n* = 50), infectious diseases (*n* = 280), thyroid dysfunction or disease (*n* = 3,346), liver and kidney disease (*n* = 3,302), cardiovascular disease (*n* = 631), history of operation (*n* = 54), using antilipemic drugs (*n* = 107) and supplements containing vitamin D (*n* = 123). Finally, a total of 8,516 participants were included (The specific exclusion process is illustrated in Supplementary Figure S1).

### 2.2 Assessment of 25(OH)D

In this study, after a 12-h fasting period, venous blood samples were collected for analysis of serum 25(OH)D using liquid chromatography-mass spectrometry [Instrument: LCMS-8050 CL (Shimadzu), Kit: 25(OH)D assay kit (liquid chromatography-tandem mass spectrometry)]. The classification of 25(OH)D levels was as follows: sufficiency (>30 ng/ml), insufficiency (20–30 ng/ml), deficiency (10–20 ng/ml), and sever deficiency (≤ 10 ng/ml) (22).

### 2.3 Assessment and definition of lipid profile

After a 12-hour fasting period, venous blood samples were collected for analysis of TC, TG, HDL-C, LDL-C using the Cobasc501 automatic biochemical analyzer from Roche Diagnostics in Germany. The calculations of nonHDL-C and RC were performed using the following formula: nonHDL-C=TC-HDL-C, RC=TC-HDL-C-LDL-C.

In this study, hypercholesterolemia (HTC) was defined as TC ≥5.2 mmol/L, hypertriglyceridemia (HTG) was defined as TG ≥1.7 mmol/L, hyper-low-density lipoprotein cholesterolemia (HLDL-C) was defined as LDL-C ≥3.4 mmol/L, and hyper-nonHDL-C (HnonHDL-C) was defined as nonHDL-C ≥4.1 mmol/L (1), hypo-high-density lipoprotein cholesterolemia (LHDL-C) was defined as HDL-C < 1.0 mmol/L in males or HDL-C < 1.3 mmol/L in females (23), and high RC (HRC) was defined as the upper quartile of RC levels.

### 2.4 Assessment of covariates

The physician-qualified conducted measurements of abdominal circumference (AC), height, and weight. Body mass index (BMI) was calculated as weight in kilograms divided by height in meters squared. Diastolic blood pressure (DBP) and systolic blood pressure (SBP) were measured using an automatic medical electronic sphygmomanometer (Omron HBP-9020). During the measurement, the interval between the two measurements was 1 to 2 min, and the average value of the two readings was recorded. After a 12-h fasting period, venous blood samples were collected for analysis of serum uric acid (UA), fasting blood glucose (FBG), alanine aminotransferase (ALT), aspartate aminotransferase (AST), γ-glutamyl transferase (GGT), globulin, alkaline phosphatase (ALP), and total bilirubin (TBil) using the Cobasc501 automatic biochemical analyzer from Roche Diagnostics in Germany.

Hypertension was defined as SBP ≥140 mmHg and/or DBP ≥90 mmHg, medical history of hypertension or self-reported hypertension (24). Type 2 diabetes mellitus (T2DM) was diagnosed as FBG ≥7 mmol/L, use of antidiabetic medication, or self-reported diagnosis of T2DM (25). Hyperuricemia was defined as serum UA levels ≥360 μmol/L in female and ≥420 μmol/L in male, or the administration of urate-lowering agents (26). Daily ultraviolet index data from 2020 to 2023 were collected from National Meteorological Information Center and Liaoning Meteorological Service. We calculated each participant's month average ultraviolet index according to time (months) of their blood collection. It was categorized into four groups: low (0–2), middle (3–6), high (7–9) and very high (≥10).

### 2.5 Statistical analysis

In this study, the normality of the data was assessed using normal Q-Q plots. Normally distributed continuous variables were described as means and standard deviation, and nonnormally distributed continuous variables were described as median and interquartile range. Categorical variables were described as frequencies and percentages. Continuous variables were compared using one-way ANOVA or the Kruskal-Wallis test, and categorical variables were compared using the chi-square test. For normally distributed continuous variables, we used Bonferroni in *post hoc* analyses to indicate differences in variables between groups. For continuous variables that did not fit the normal distribution, we used Dunn test in *post hoc* analyses to indicate differences in variables between groups (Supplementary Tables S2, S3). In addition, we used ANCOVA to analyze the effect of 25(OH)D on lipid profiles, controlling for age, sex, and BMI (Supplementary Table S4).

Logistic regression model was used to explore the association between 25(OH)D and lipid profile, while calculating odds ratio (OR) and 95% confidence intervals (CI). Model 1 did not account for covariates. Model 2 was adjusted for sex (categorical), age (continuous), and the season (categorical). Model 3 was adjusted for covariates including sex (categorical), age (continuous), season (categorical), and BMI (continuous). The additional model 4 was adjusted for covariates including sex (categorical), age (continuous), season (categorical), hypertension (categorical), diabetes (categorical), hyperuricemia (categorical), BMI (continuous), AC (continuous), ALT, AST, Globulin, GGT, ALP, Tbil, Urea, and UA (all continuous). In this study, the variance inflation factor (VIF) was used to measure the degree of multicollinearity in multiple linear regression models, and the regression models all had VIFs < 4, indicating that there was not a high degree of multicollinearity in the covariates of the regression model (Supplementary Table S6). The high multicollinearity was shown between season and UV index (VIF > 4), therefore only season was used as a covariate in the adjusted models. Dose-response relationship between 25(OH)D and lipid profile was analyzed using restricted cubic spline (RCS) fitting logistic regression. The application of RCS, which are smooth functions, has been extensively employed in academic research for analyzing nonlinear associations between variables and outcomes (27, 28). The selection of nodes is the most crucial aspect in RCS, and the optimal fitting degree would be 4 nodes (29). We placed the nodes at the 27.5th, 50th, 67.5th, and 95th percentiles of 25(OH)D levels based on the data situation, without setting any reference points. The 27.5th, 50th, 67.5th, and 95th percentile values of vitamin D levels were 12.92 ng/ml, 16.87 ng/ml, 20.55 ng/ml, and 31.22 ng/ml. The piecewise linear regression model was used to establish a smooth curve, aiming to enhance the precision of determining appropriate 25(OH)D cut-off points corresponding to lipid profile.

The statistical analyses were conducted using STATA SE (version 15.0) and R software (version 4.2.3). The statistical significance was determined when the two-sided *P* < 0.05.

## 3 Results

### 3.1 Characteristics of the participants

The characteristics of the study participants were presented in Table 1. Among the 8,516 participants, slightly more were women, about 2/3 (65.0%) in total were 25(OH)D deficient (25(OH)D < 20 ng/mL), and mean vit D levels were in the range of deficiency. Among women, 39.4%, 26.3%, 27.8%, 56.8%, 51.6%, and 20.2% had abnormal levels of TC, TG, HDL-C, LDL-C, nonHDL-C, and RC, respectively. Among men, 41.9%, 45.6%, 21.7%, 72.3%, 68.4%, and 29.3% had abnormal levels of TC, TG, HDL-C, LDL-C, nonHDL-C, and RC, respectively. Interestingly, we found that when there was a deficiency (10–20 ng/ml) or severe deficiency (≤ 10 ng/ml) of 25(OH)D, the levels of TC, TG, LDL-C, nonHDL, RC, AC, BMI, ALT, AST, Globulin, GGT, ALP, Tbil, and Urea were lower than those in the participant population with 25(OH)D >20 ng/ml (*P* < 0.001), while the levels of HDL-C were higher than that in the participant population with 25(OH)D >20 ng/ml (*P* < 0.001) (Table 1). Post hoc ANOVA analysis showed that compared with vitamin D sufficient group, vitamin D severely deficient group had a better lipid profile, vitamin D insufficient group had mostly a worse lipid profile, while vitamin D moderately deficient subjects were not different. *Post hoc* ANCOVA analysis showed that after adjustment for age, sex, and BMI, compared with vitamin D deficient group, the differences in lipid levels were still significant for vitamin D severely deficient group. In addition, the 25(OH)D concentrations were higher in males, 50–74 years old participants, during summer/autumn and high/very high UV index (in Table 1, Supplementary Tables S1–S3, Supplementary Figure S2).

**Table 1:** Characteristics of the participants according to 25(OH)D concentrations.

**Variables**	**25(OH)D concentrations (ng/ml)**					**F/H/χ^2^ value**	***P* value**
	**Total**	>**30**	**20–30**	**10–20**	≤ **10**		
*N* (%)	8,516 (100)	560 (6.6)	2,416 (28.4)	4,478 (52.6)	1,062 (12.4)		
Sex			^***^	^***^	^***^	6.8	**< 0.001**
Female *N* (%)	4,766 (56.0)	177 (3.7)	979 (20.6)	2,761 (57.9)	849 (17.8)		
Male *N* (%)	3,750 (44.0)	383 (10.2)	1,437 (38.3)	1,717 (45.8)	213 (5.7)		
Age (years)	41 (34–50)	49 (38–56)	44 (36–52)^***^	40 (34-49) ^***^	35 (29-42) ^***^	**6.3**	**< 0.001**
Age (years)			^***^	^***^	^***^	**4.3**	**< 0.001**
18–50 N (%)	6,360 (74.7)	292 (4.6)	1,593 (25.1)	3,514 (55.2)	961 (15.1)		
50–74 N (%)	2,156 (25.3)	268 (12.4)	823 (38.2)	964 (44.7)	101 (4.7)		
Season			^*^	^***^	^***^	9.5	**< 0.001**
Spring *N* (%)	1,186 (13.9)	43 (3.6)	197 (16.6)	716 (60.4)	230 (19.4)		
Summer *N* (%)	2,911 (34.2)	231 (7.9)	1,043 (35.8)	1,440 (49.5)	197 (6.8)		
Autumn *N* (%)	2,903 (34.1)	261 (9.0)	987 (34.0)	1,460 (50.3)	195 (6.7)		
Winter *N* (%)	1,516 (17.8)	25 (1.6)	189 (12.5)	862 (56.9)	440 (29.0)		
Ultraviolet index			^*^	^***^	^***^	9.4	**< 0.001**
Low *N* (%)	1,899 (22.3)	49 (2.6)	297 (15.6)	1,109 (58.4)	444 (23.4)		
Middle *N* (%)	1,197 (14.1)	40 (3.3)	169 (14.1)	699 (58.4)	289 (24.2)		
High *N* (%)	2,509 (29.4)	240 (9.6)	907 (36.1)	1,230 (49.0)	132 (5.3)		
Very high *N* (%)	2,911 (34.2)	231 (7.9)	1,043 (35.8)	1,440 (49.5)	197 (6.8)		
25(OH)D (ng/ml)	18.0 ± 7.1	33.7 ± 2.8	24.1 ± 2.8	15.0 ± 2.8	8.3 ± 1.2	17.8	**< 0.001**
AC (cm)	83.1 ± 0.1	86.2 ± 0.4	85.8 ± 0.2	82.5 ± 0.15^***^	78.7 ± 0.3^***^	14.9	**< 0.001**
BMI (kg/m^2^)	23.7 ± 0.1	24.4 ± 0.2	24.4 ± 0.1	23.6 ± 0.1^***^	22.2 ± 0.2^***^	7.4	**< 0.001**
TC (mmol/L)	5.04 ± 0.01	5.10 ± 0.04	5.12 ± 0.03^***^	5.02 ± 0.01^#^	4.89 ± 0.02^***###^	11.3	**< 0.001**
TG (mmol/L)	1.37 (0.96–1.97)	1.46 (1.06–2.03)	1.47 (1.05–2.08)	1.34 (0.94–1.93)^**^	1.20 (0.87–1.89)^***###^	9.0	**< 0.001**
HDL-C (mmol/L)	1.38 ± 0.01	1.35 ± 0.01	1.33 ± 0.01^***^	1.39 ± 0.01^*^	1.45 ± 0.01^***###^	13.5	**< 0.001**
LDL-C (mmol/L)	2.90 ± 0.01	2.97 ± 0.03	2.98 ± 0.02^***^	2.88 ± 0.01^##^	2.76 ± 0.02^***###^	6.8	**< 0.001**
nonHDL-C (mmol/L)	3.66 ± 0.01	3.76 ± 0.04	3.79 ± 0.02^***#^	3.64 ± 0.01^**##^	3.44 ± 0.02^***###^	7.3	**< 0.001**
RC (mmol/L)	0.76 ± 0.01	0.79 ± 0.01	0.81 ± 0.01^###^	0.76 ± 0.01^#^	0.68 ± 0.01^***##^	8.8	**< 0.001**
ALT (U/L)	18.6 (13.5–28.2)	21.1 (15.9–30.7)	20.7 (15.2–31.0)	18.0 (13.1–27.4) ^***^	15.3 (11.2–22.3) ^***^	8.3	**< 0.001**
AST (U/L)	19.7 (16.7–23.8)	21.2 (18.2–25.7)	20.7 (17.6–25.0)	19.3 (16.4–23.4) ^***^	18.4 (15.9–21.5) ^***^	12.4	**< 0.001**
Globulin (g/L)	27.7 ± 0.0	27.9 ± 0.1	27.6 ± 0.1^*^	27.8 ± 0.1^***^	27.8 ± 0.1^**^	6.6	**< 0.001**
GGT (U/L)	16.7 (11.5–26.9)	20.7 (14.4–30.9)	19.5 (13.4–30.7)	15.8 (11.2–25.7)^***^	12.9 (9.7–19.7)^***^	8.9	**< 0.001**
ALP (U/L)	64.0 (53.0–77.8)	68.0 (57.0–81.0)	66.8 (55.7–80.0)	63.5 (52.3–76.7)^***^	60.1 (50.1–72.7)^***^	11.9	**< 0.001**
Tbil (μmol/L)	13.8 (11.0–17.6)	14.1 (11.3–18.3)	14.5 (11.6–18.5)	13.6 (10.9–17.3)^*^	12.9 (10.1–16.5)^***^	10.5	**< 0.001**
Urea (mmol/L)	4.9 (4.1–5.7)	5.3 (4.6–6.1)	5.0 (4.4–5.9)^***^	4.8 (4.1–5.6)^***^	4.4 (3.7–5.1)^***^	5.4	**< 0.001**
Hypertension				^***^	^***^	8.5	**< 0.001**
Yes *N* (%)	1,229 (14.4)	112 (9.1)	428 (34.8)	581 (47.3)	108 (8.8)		
No *N* (%)	7,287 (85.6)	448 (6.1)	1,988 (27.3)	3,897 (53.5)	954 (13.1)		
Diabetes				^***^	^***^	9.8	**< 0.001**
Yes *N* (%)	381 (4.5)	36 (9.5)	151 (39.6)	170 (44.6)	24 (6.3)		
No *N* (%)	8,135 (95.5)	524 (6.4)	2,265 (27.8)	4,308 (53.0)	1,038 (12.8)		
Hyperuricemia				^***^	^***^	16.6	**< 0.001**
Yes *N* (%)	2,440 (28.7)	205 (8.4)	874 (35.8)	1,180 (48.4)	181 (7.4)		
No *N* (%)	6,076 (71.3)	355 (5.8)	1,542 (25.4)	3,298 (54.3)	881 (14.5)		

25(OH)D, 25-hydroxyvitamin D; AC, Abdominal circumference; BMI, Body mass index; DBP, Diastolic blood pressure; FBG, Fasting blood glucose; TC, Total cholesterol; TG, Triglyceride; HDL-C, High density lipoprotein cholesterol; LDL-C, Low Density Lipoprotein; nonHDL, non-high-density lipoprotein cholesterol; RC, Remnant cholesterol; ALT, Alanine aminotransferase; AST, Aspartate aminotransferase; GGT, γ-glutamyl transferase; ALP, Alkaline phosphatase; Tbil, Total bilirubin.

Bold font indicates statistically significant differences (P < 0.05).

^*^, ^**^, ^***^ used to label the significant comparisons with vitamin D sufficient group (> 30 nmol/L), meaning p < 0.05, p < 0.01, p < 0.001, respectively.

^#^, ^##^, ^###^ used to label the significant comparisons with vitamin D sufficient group (> 30 nmol/L) after adjustment for sex, age, and BMI, meaning p < 0.05, p < 0.01, p < 0.001, respectively.

### 3.2 25(OH)D deficiency was associated with an increased prevalence of abnormal lipid profile

In the model adjusted for sex, age, season, and BMI (Model 3, Table 2), compared to individuals with sufficient levels of 25(OH)D (>30 ng/ml), those with insufficient or deficient 25(OH)D levels (30–20 ng/ml, 10–20 ng/ml, or < 10 ng/ml) had significantly higher risks (ORs) for all examined dyslipidemia types (Table 2). The non-significant but still increased risk was only shown for HTG and HRC among those with severe vitamin D deficiency (< 10 ng/ml) (Table 2). In the adjusted model (Model 3), which accounted for sex, age, season and BMI, the highest risk was noted among vitamin D moderately deficient, then among vitamin D insufficient subjects, while those with severe vitamin D deficiency had the least increased significant risk, which is an interesting finding. Interestingly, in the non-adjusted model (Model 1), only the lower risk for all dyslipidemia types was shown among those with vitamin D severe deficiency, while in Model 2, adjusted only for sex, age, and season, only among those with vitamin D insufficiency/moderate deficiency the risk was increased and only for some dyslipidemia types (HTC, LHDL-C, HnonHDL-C, HRC). The correction for BMI, therefore, strengthened the level of association of vitamin D deficiency/insufficiency with dyslipidemia, and further correction for other cardiometabolic traits in the fully adjusted additional model (Model 4) additionally strengthened the level of association (Supplementary Table S7).

**Table 2:** The associations between 25(OH)D and lipid profile.

	**Model 1**		**Model 2**		**Model 3**	
	**OR (95%CI)**	**P value**	**OR (95%CI)**	**P value**	**OR (95%CI)**	**P value**
**HTC**						
25(OH)D suffciency	1		1		1	
25(OH)D insufficiency	1.111 (0.922, 1.339)	0.267	1.240 (1.024, 1.501)	**0.027**	1.305 (1.103, 1.545)	**0.002**
25(OH)D deficiency	0.937 (0.784, 1.120)	0.476	1.154 (0.957, 1.393)	0.134	1.356 (1.189, 1.673)	**< 0.001**
Severe 25(OH)D deficiency	0.659 (0.533, 0.814)	**< 0.001**	0.934 (0.741, 1.176)	0.562	1.219 (1.050, 1.415)	**0.009**
**HTG**						
25(OH)D suffciency	1		1		1	
25(OH)D insufficiency	1.039 (0.859, 1.256)	0.694	1.154 (0.949, 1.402)	0.151	1.196 (1.003, 1.726)	**0.025**
25(OH)D deficiency	0.822 (0.686, 0.986)	**0.035**	1.048 (0.865, 1.270)	0.632	1.263 (1.057, 1.438)	**0.006**
Severe 25(OH)D deficiency	0.762 (0.615, 0.944)	**0.013**	1.086 (0.857, 1.377)	0.493	1.089 (0.859, 1.382)	0.376
**HLDL-C**						
25(OH)D suffciency	1		1		1	
25(OH)D insufficiency	1.034 (0.851, 1.257)	0.734	1.177 (0.964, 1.437)	0.109	1.193 (1.002, 1.438)	**0.049**
25(OH)D deficiency	0.880 (0.731, 1.060)	0.179	1.207 (0.993, 1.466)	0.059	1.226 (1.097, 1.536)	**0.012**
Severe 25(OH)D deficiency	0.608 (0.491, 0.752)	**< 0.001**	0.986 (0.782, 1.245)	0.908	1.331 (1.018, 1.740)	**0.037**
**LHDL-C**						
25(OH)D suffciency	1		1		1	
25(OH)D insufficiency	1.045 (0.824, 1.325)	0.717	1.229 (0.981, 1.539)	0.073	1.308 (0.998, 1.769)	0.057
25(OH)D deficiency	1.138 (0.902, 1.437)	0.274	1.298 (1.041, 1.618)	**0.021**	1.323 (1.109, 1.684)	**0.004**
Severe 25(OH)D deficiency	1.218 (0.922, 1.609)	0.165	1.294 (0.995, 1.682)	0.055	1.380 (1.107, 1.753)	**0.023**
**HnonHDL**						
25(OH)D suffciency	1		1		1	
25(OH)D insufficiency	1.128 (0.932, 1.364)	0.216	1.339 (1.100, 1.630)	**0.004**	1.478 (1.132, 1.983)	**< 0.001**
25(OH)D deficiency	0.831 (0.693, 0.996)	**0.045**	1.243 (1.026, 1.506)	**0.026**	1.356 (1.174, 1.892)	**< 0.001**
Severe 25(OH)D deficiency	0.530 (0.430, 0.653)	**< 0.001**	1.021 (0.811, 1.285)	0.861	1.089 (1.007, 1.498)	**0.048**
**HRC**						
25(OH)D suffciency	1		1		1	
25(OH)D insufficiency	1.168 (0.949, 1.437)	0.142	1.378 (1.115, 1.704)	**0.003**	1.398 (1.174, 1.793)	**< 0.001**
25(OH)D deficiency	0.842 (0.689, 1.029)	0.093	1.249 (1.012, 1.542)	**0.038**	1.425 (1.203, 1.841)	**< 0.001**
Severe 25(OH)D deficiency	0.497 (0.387, 0.640)	**< 0.001**	0.996 (0.759, 1.308)	0.977	1.013 (0.769, 1.333)	0.929

Model 1 did not account for variables. Model 2 was adjusted for sex (categorical), age (continuous), and the season (categorical). Model 3 was adjusted for covariates including sex (categorical), age (continuous), season (categorical), and BMI (continuous).

25(OH)D, 25-hydroxyvitamin D; OR, odds ratio; CI, confidence interval. HTC, hypercholesterolemia; HTG, hypertriglyceridemia; LHDL-C, hypo-high-density lipoprotein cholesterolemia; HLDL-C, hyper-low-density lipoprotein cholesterol cholesterolemia; HnonHDL, hyper-non-high-density lipoprotein cholesterol; HRC, high remnant cholesterol.

Bold font indicates statistically significant differences (P < 0.05).

### 3.3 Nonlinear relationship between 25(OH)D and lipid profile and the determination of cut-off points for 25(OH)D

**Figure 1:**
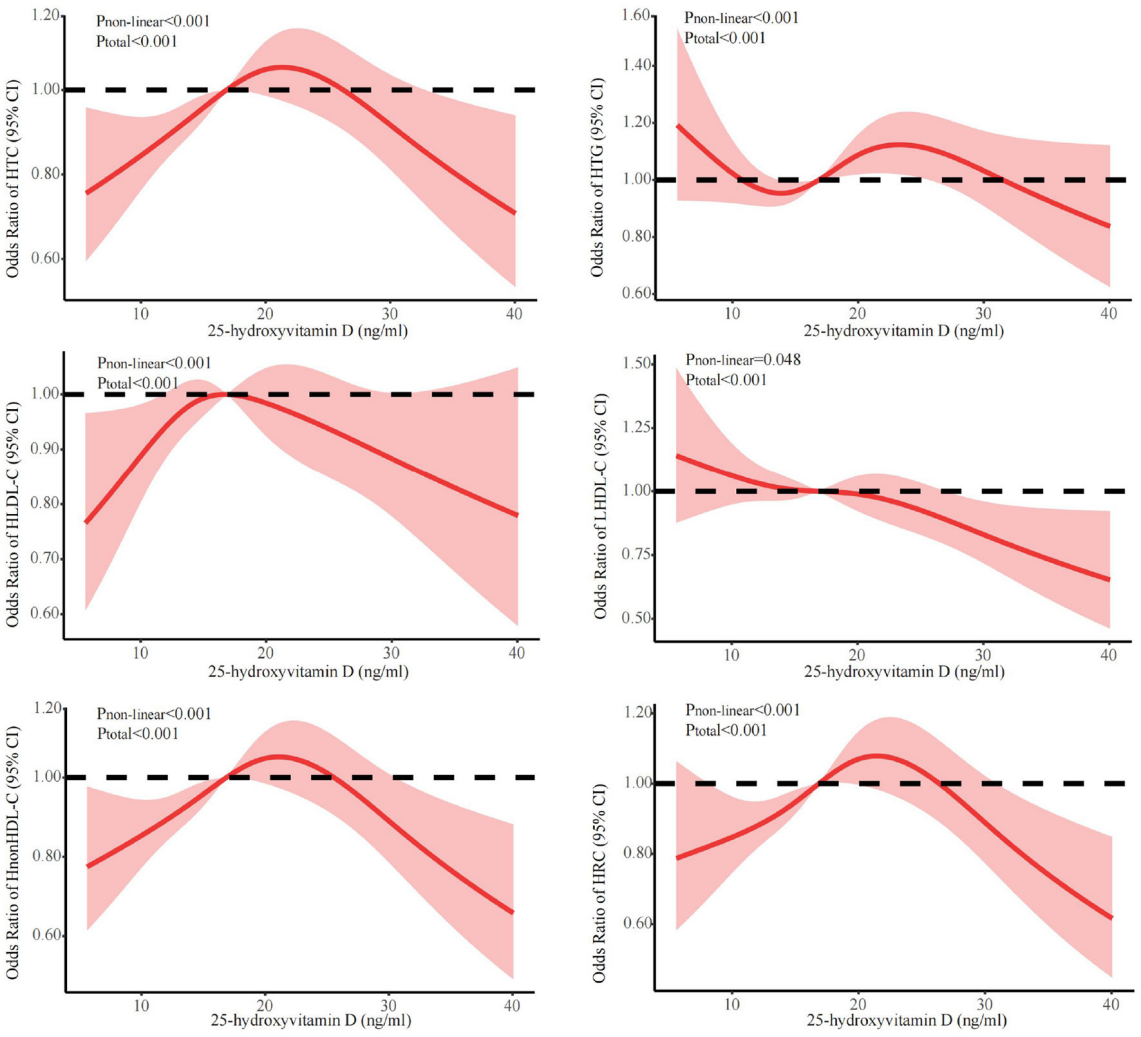
Dose-response relationship between 25(OH)D and lipid profile. Adjusted for covariates including sex (categorical), age (continuous), season (categorical), and BMI (continuous). 25(OH)D, 25-hydroxyvitamin D; CI, confidence interval; HTC, hypercholesterolemia; HTG, hypertriglyceridemia; LHDL-C, hypo-high-density lipoprotein cholesterolemia; HLDL-C, hyper-low-density lipoprotein cholesterol cholesterolemia; HnonHDL, hyper-non-high-density lipoprotein cholesterol; HRC, high remnant cholesterol.

Figure 1 showed the associations between 25(OH)D concentration and lipid profile after adjustments for sex, age, season, and BMI. The prevalence of HTG initially decreased, followed by an increase and subsequent decrease with increasing 25(OH)D concentration. Conversely, the prevalence of HTC, HLDL-C, HnonHDL, and HRC showed an initial increase followed by a decrease. The prevalence of LHDL-C showed a more linear decrease. Further, we discovered that falling below the cut-off points for 25(OH)D resulted in abnormal lipid profile. The inflection points were 23.7 ng/ml for HTC, 24.3 ng/ml for HTG, 18.5 ng/ml for HLDL-C, 23.3 ng/ml for LHDL-C, 23.3 ng/ml for HnonHDL, and 24.3 ng/ml for HRC. Below the cut-off point, for every one-unit decrease in 25(OH)D level, there was a corresponding decrease of 2.0% in the risk for elevated HTC, 1.6% in HTG, 2.8% in HLDL-C, 4.6% in HnonDL-C, and 4.1% in HRC (in Table 3). For vitamin D levels below these inflection points, the risk is significantly increased for dyslipidemia, except LHDL-C (Table 3).

**Table 3:** The cut-off value for 25(OH)D in an abnormal lipid profile.

	**25(OH)D change point, ng/ml (95%CI)**	**OR below change point (95%CI)**	**OR above change point (95%CI)**
HTC	23.7 (21.7, 25.7)	1.020 (1.006, 1.033)^*^	0.989 (0.969, 1.001)
HTG	24.3 (22.5, 26.1)	1.016 (1.011, 1.023)^*^	0.974 (0.945, 1.004)
HLDL-C	18.5 (18.2, 18.8)	1.028 (1.022, 1.034)^*^	0.991 (0.971, 1.011)
LHDL-C	23.3 (17.3, 29.3)	0.988 (0.971, 1.005)	0.976 (0.953, 0.997)^*^
HnonHDL	23.3 (21.4, 25.2)	1.046 (1.033, 1.059)^*^	0.977 (0.958, 0.997)^*^
HRC	24.3 (22.4, 26.2)	1.041 (1.035, 1.047)^*^	0.963 (0.936, 0.991)^*^

25(OH)D, 25-hydroxyvitamin D; OR, odds ratio; CI, confidence interval; HTC, hypercholesterolemia; HTG, hypertriglyceridemia; LHDL-C, hypo-high-density lipoprotein cholesterolemia; HLDL-C, hyper-low-density lipoprotein cholesterol cholesterolemia; HnonHDL, hyper-non-high-density lipoprotein cholesterol; HRC, high remnant cholesterol.

Adjusted for covariates including sex (categorical), age (continuous), season (categorical), and BMI (continuous).

^*^used to mean p < 0.05, respectively.

### 3.4 The stratified analyses of the nonlinear relationships between 25(OH)D and lipid profile

**Figure 2:**
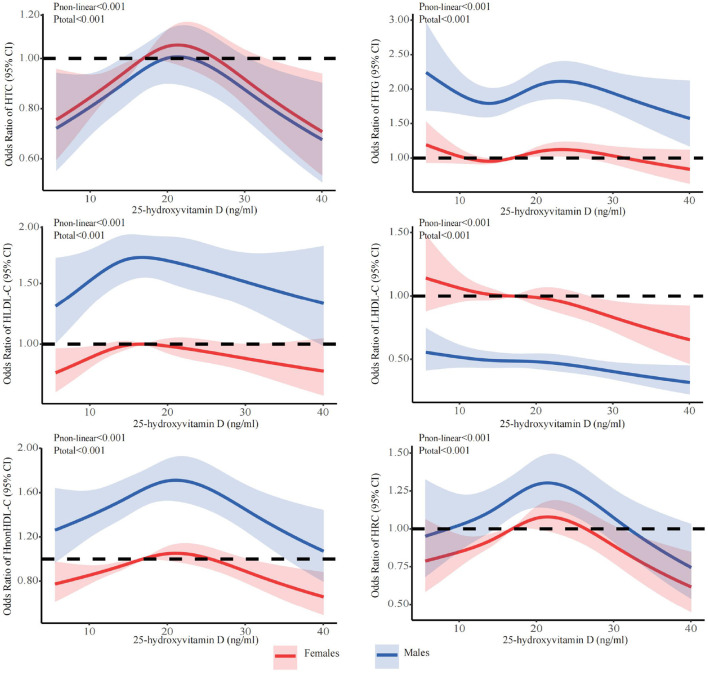
Dose-response relationship between 25-hydroxyvitamin D and the risk for specific dyslipidemias stratified by sex. Adjusted for covariates including sex (categorical), age (continuous), season (categorical), BMI (continuous). CI, confidence interval; HTC, hypercholesterolemia; HTG, hypertriglyceridemia; LHDL-C, hypo-high-density lipoprotein cholesterolemia; HLDL-C, hyper-low-density lipoprotein cholesterol cholesterolemia; HnonHDL-C, hyper-non-high-density lipoprotein cholesterol; HRC, high remnant cholesterol.

**Figure 3:**
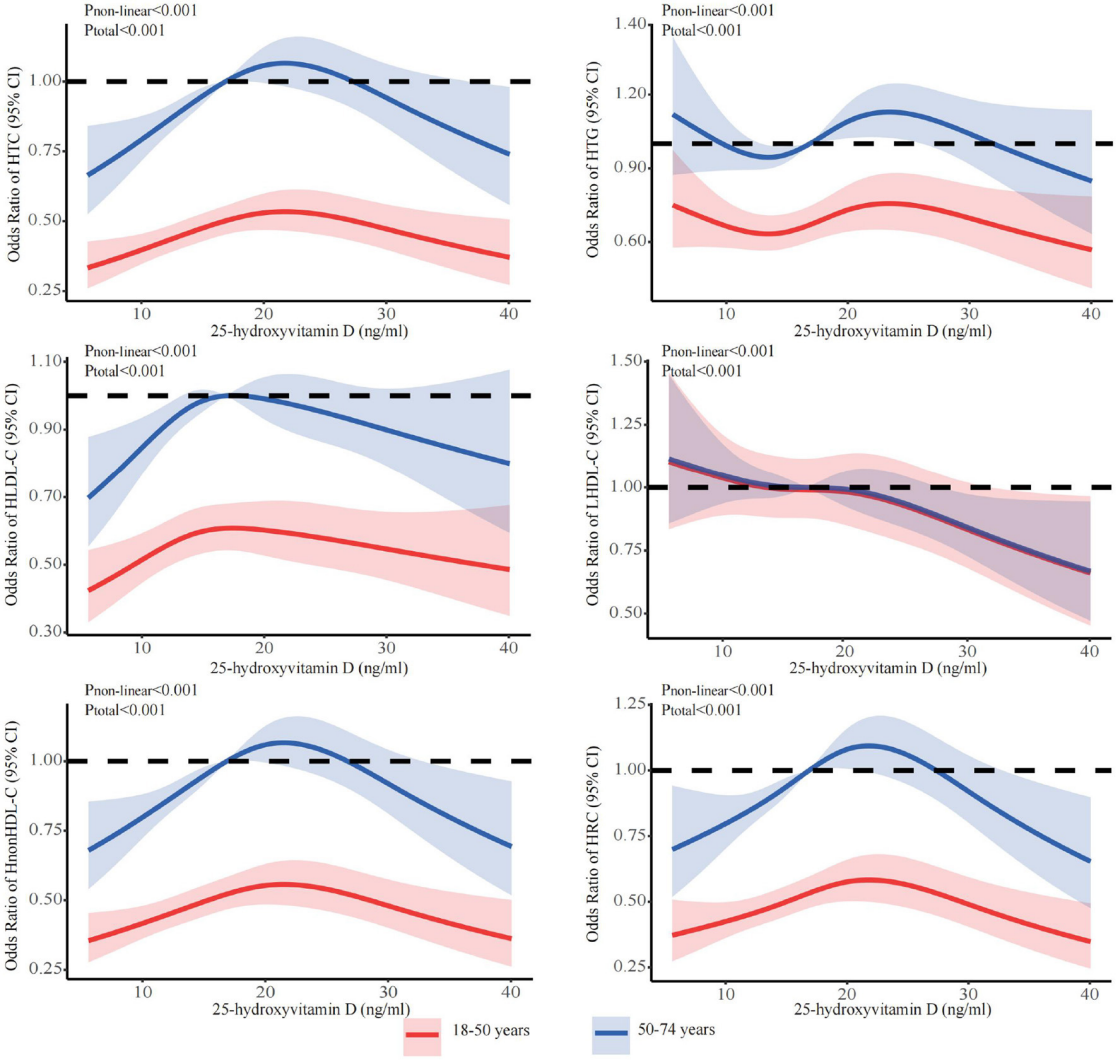
Dose-response relationship between 25-hydroxyvitamin D and the risk for specific dyslipidemias stratified by age. Adjusted for covariates including sex (categorical), age (continuous), season (categorical), BMI (continuous). CI, confidence interval; HTC, hypercholesterolemia; HTG, hypertriglyceridemia; LHDL-C, hypo-high-density lipoprotein cholesterolemia; HLDL-C, hyper-low-density lipoprotein cholesterol cholesterolemia; HnonHDL-C, hyper-non-high-density lipoprotein cholesterol; HRC, high remnant cholesterol.

**Figure 4:**
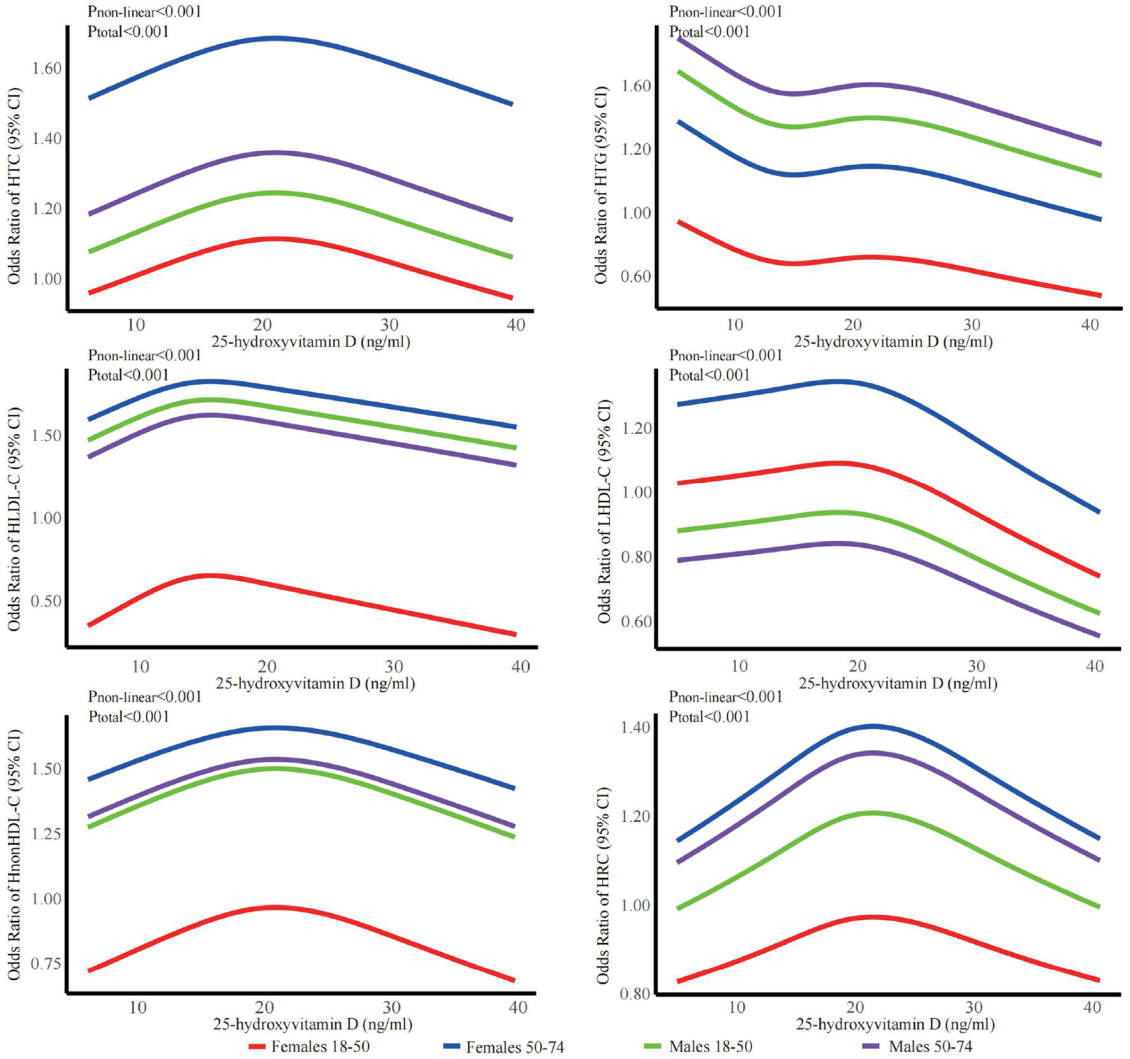
Dose-response relationship between 25-hydroxyvitamin D and the risk for specific dyslipidemias stratified by sex + age. Adjusted for covariates including sex (categorical), age (continuous), season (categorical), BMI (continuous). CI, confidence interval; HTC, hypercholesterolemia; HTG, hypertriglyceridemia; LHDL-C, hypo-high-density lipoprotein cholesterolemia; HLDL-C, hyper-low-density lipoprotein cholesterol cholesterolemia; HnonHDL-C, hyper-non-high-density lipoprotein cholesterol; HRC, high remnant cholesterol.

**Figure 5:**
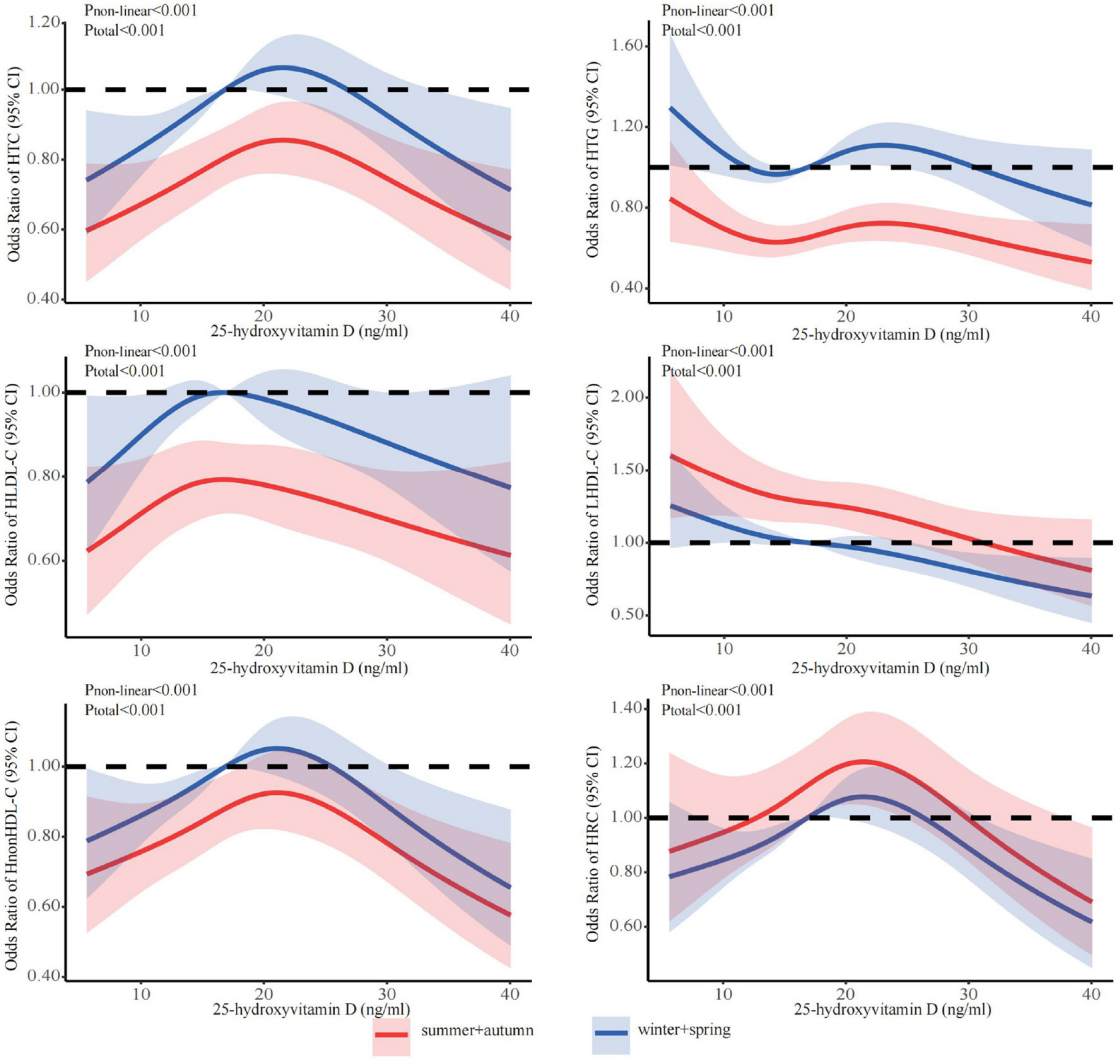
Dose-response relationship between 25-hydroxyvitamin D and the risk for specific dyslipidemias stratified by season. Adjusted for covariates including sex (categorical), age (continuous), season (categorical), BMI (continuous). CI, confidence interval; HTC, hypercholesterolemia; HTG, hypertriglyceridemia; LHDL-C, hypo-high-density lipoprotein cholesterolemia; HLDL-C, hyper-low-density lipoprotein cholesterol cholesterolemia; HnonHDL-C, hyper-non-high-density lipoprotein cholesterol; HRC, high remnant cholesterol.

**Figure 6:**
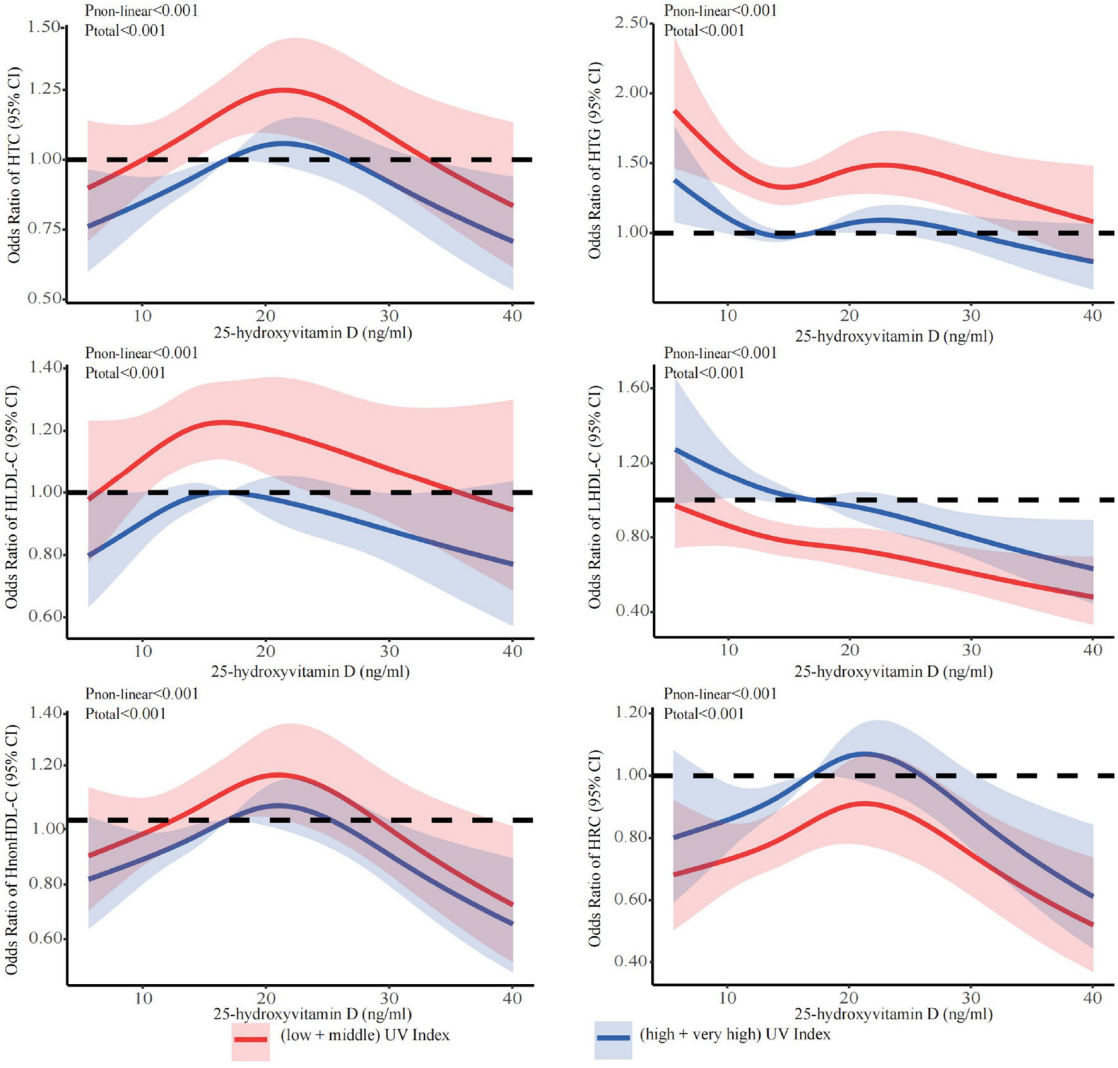
Dose-response relationship between 25-hydroxyvitamin D and the risk for specific dyslipidemias stratified by ultraviolet index. Adjusted for covariates including sex (categorical), age (continuous), season (categorical), BMI (continuous). CI, confidence interval; HTC, hypercholesterolemia; HTG, hypertriglyceridemia; LHDL-C, hypo-high-density lipoprotein cholesterolemia; HLDL-C, hyper-low-density lipoprotein cholesterol cholesterolemia; HnonHDL-C, hyper-non-high-density lipoprotein cholesterol; HRC, high remnant cholesterol.

The stratified analyses (Figures 2–6) showed that a higher risk of abnormal lipid profile under equivalent 25(OH)D concentrations was observed among males (Figure 2), 191 individuals aged 50–74 years (Figure 3), during the winter/spring period (Figure 5), and under low/middle ultraviolet index environments (Figure 6). More importantly, further stratification of sex categories by age has shown that the risk for dyslipidemia was the highest in women aged 50–74 years, except for HTG, which was the highest among men aged 50–74 years (Figure 4). The lowest risk was noted in women aged 18–50 years, except for LHDL-C, where the risk was still higher compared to men. The results were not very different if model 4 was applied (Supplementary Figures S3–S7).

## 4 Discussion

In this study, nonlinear inverse U-shaped associations were observed between 25(OH)D levels and abnormal lipid profiles, except for LHDL-C, where more an inverse linear association was observed. The level of 25(OH)D below the inflection points showed positive correlations with the risks of abnormal lipid profile, with inflection points observed at 23.7 ng/ml for HTC, 24.3 ng/ml for HTG, 18.5 ng/ml for HLDL-C, 23.3 ng/ml for LHDL-C, 23.3 ng/ml for HnonHDL-C, and 24.3 ng/ml for HRC. The stratified analyses showed that the risk for most dyslipidemias related to deficiency of 25(OH)D was particularly increased among females aged 50-74 (except for hypertriglyceridemia, where the highest risk was among men aged 50–74 years), during winter/spring or under low/middle ultraviolet index environments.

The prevalence of vitamin D deficiency in adults varies due to regional and ethnic differences. The incidence of vitamin D deficiency ranges between 20% and 60% in various European countries (30). In Asia, the overall prevalence of vitamin D deficiency among the general population is estimated at 75% (31). In China, the incidence of vitamin D deficiency among residents is estimated to be 87.1% (32).

Previous studies have demonstrated that maintaining 25(OH)D concentrations between 25 and 60 ng/ml significantly decreases all-cause mortality rates, lowers the risk of cancer, and reduces the incidence of diseases such as T2DM and hypertension (33, 34). The evidence indicated that the body should maintain a minimum level of 30 ng/ml of 25(OH)D in order to support musculoskeletal health (22, 35). Several studies have shown that vitamin D supplementation may be beneficial in improving dyslipidemia in individuals with vitamin D deficiency (36–39). In investigating the association between 25(OH)D and blood lipid, it was observed that a 25(OH)D level below 20 ng/ml increased the risk of dyslipidemia (33, 40–43), which is consistent with our findings (represented as ORs in Table 3). Above this value, the risk gradually decreased in our study. The results of our study indicated an inverse U-shaped relationship between 25(OH)D and abnormal lipid profile. The 25(OH)D levels above 20 ng/ml appeared to have a protective effect on lipid profile. The positive correlation between 25(OH)D and the risk of dyslipidemia in the range of 25(OH)D < 20 ng/ml found by RCS curves cannot be easily explained, but logistic regression models showed that there is still an increased risk compared with the vitamin D sufficient group, even in those with severe deficiency. We also found that the risk of abnormal lipid profile was lower in participants with severe 25(OH)D deficiency (≤ 10 ng/ml) compared to those with deficiency (10–20 ng/ml) and insufficiency (20–30 ng/ml). The reason for such findings can be the lower BMI and abdominal circumference in this group of participants. Additionally, there can be other possible explanations, which require further exploration.

In the synthesis of cholesterol from acetyl-CoA, the rate-limiting step is the conversion of HMG-CoA to mevalonate by HMG-CoA reductase, after which follow series of reactions that involve >20 steps. The final step in the Kandutsch-Russell pathway of cholesterol synthesis is a conversion 7DHC to cholesterol by 7-dehydrocholesterol reductase (DHCR7) through the reduction of the C (7, 8) double bond, and this can occur ubiquitously in the body. However, in the skin, previtamin D3 is formed from 7DHC by the cleavage of the C (9, 10) bond through exposure to photons of ultraviolet B (UV-B) light, thus reducing the substrate for cholesterol synthesis (44). Therefore UV-B light, by conversion of 7DHC to vitamin D, may reduce the substrate for skin cholesterol synthesis. Additionally, UV-B light stimulates the production of the active form of vitamin D, 1,25(OH)D in human skin keratocytes (keratinocytes express both 25-hydroxylase and 1α-hydroxylase, and UV-B stimulates 1α-hydroxylase expression) and reduces expression of DHCR7 (45). It was also shown in human keratinocytes that cholesterol and its precursors induce proteasomal degradation of DHCT7, thus reducing its activity and causing the increase in vitamin D synthesis (46). It was shown in cultured human skin fibroblasts, transformed human liver cells, mouse intestinal epithelial cells, and mouse peritoneal macrophages, that vitamin D and its hydroxylated metabolites inhibited cholesterol synthesis. All hydroxylated derivatives (but not vitamin D) inhibited 14a-lanosterol demethylase. Additionally, vitamin D and 25(OH)D, but not 1,25(OH)D, inhibited HMG-CoA reductase activity in a concentration-dependent manner. Interestingly, in cultured human skin fibroblasts, 1,25(OH)D had a biphasic effect on HMG-CoA reductase, with inhibiting at low concentrations and restoring to control values at high concentrations, while in transformed human liver cells it strongly stimulated HMG-CoA reductase activity, which opposed the effect of 25(OH)D. Additionally, it was shown in human glioma cell lines that vitamin D3 but not 1,25(OH)D inhibits the expression of DHCR7, which leads to the inhibition of cholesterol synthesis and the accumulation of 7-DHC and other sterol intermediates. Therefore the effect of vitamin D and its metabolites can vary between different tissues, and the effect of vitamin D, 25(OH)D, and its active form 1,25(OH)D can be significantly different (47).

Interestingly, in an epidemiological study of 307 apparently healthy 40–60 years old Indian men (48), serum DHCR7 levels were significantly lower in subjects at higher daily sun exposure, and there was a negative association between serum 25(OH)D and DHCR7 levels at moderate and higher sunlight exposure (but not at lower sunlight exposure). At the same time, it was shown that while at moderate sunlight exposure (1–2 h/d), there was no significant association between serum 25(OH)D and HDL-C levels, at lower sunlight exposure (< 1 h/d), there was a positive association, and, in contrast, at higher sunlight exposure (>2 h/d), there was a negative association (48), which is in alignment with our findings that the risk for LHDL-C was the highest during summer/autumn and very high and high UV index.

However, some experimental data did not manage to confirm the significant effect of UV light on the serum lipid levels: the whole body UV-A and UV-B radiation twice weekly for 12 weeks with suberythemal doses did not make significant differences in any lipoproteins or apolipoprotein levels between the treated and control, despite increasing significantly vitamin D levels in the treated group (49). Nevertheless, in another experimental study in Indian men, there was a decrease in TC, HDL-C, and LDL-C with moderately increasing daily sunlight exposure, while, in contrast, vitamin D supplementation with oral cholecalciferol 1,000 IU/day led to the opposite effect (particularly on TC and HDL-C) (50). Both interventions significantly increased the vitamin D serum levels compared with the control group, indicating that there can be a difference between the effect of exogenous and endogenous vitamin D levels on different cholesterol subfractions. In general, the studies on the effect of vitamin D supplementation on lipid levels revealed inconsistent findings (41).

25(OH)D was found to inhibit the expression of adipogenic transcription factor genes, thereby significantly reducing lipid accumulation and promoting adipocyte apoptosis. Nevertheless, adipose tissue has the capacity to sequester vitamin D, thereby exerting an additional cumulative effect that results in a reduction in the amount of vitamin D entering the circulation (51). Additionally, there is an interplay between vitamin D and cortisol, with vitamin D downregulating cortisol receptors and thus diminishing its activity (52, 53). Moreover, there is an interaction with other steroid hormones and nuclear receptors involved in metabolism (54). 25(OH)D enhances lipoprotein lipase activity and gene expression in muscle and adipose tissue, accelerating clearance of lipoprotein particles in the circulation and lowering in TG concentrations (55–59). 25(OH)D also enhances intracellular calcium ion levels in hepatocytes, which contributes to reverse cholesterol transport (60, 61).

The people aged 50–74 years had higher levels of 25(OH)D compared to those aged 18–50 years (which is in contrast with other studies, which show lower levels of vitamin D in older adults, connected with lower exposure to sun, decreased skin production of vitamin D, and lower intake) (62). However, they were more likely to have dyslipidemia at the given serum concentration of vitamin 25(OH)D. This is in accordance with the higher prevalence of dyslipidemia in general with aging and related changes in metabolism, insulin sensitivity hormones, hormonal and body composition. Another potential explanation is that with aging the activation and function of 25(OH)D decrease (connected with a lower concentration of vitamin D receptor and lower kidney 21-hydroxylation), which further decreases the regulation of lipid metabolism and leads to an increased prevalence of dyslipidemia (63).

Although 25(OH)D levels were higher in men than in women in the present study, the risk of dyslipidemia at given vitamin D serum concentrations was higher in men, when the whole sample was stratified only by sex, with the exception of LHDL-C and HRC (“nonLDL/nonHDL-cholesterol”), where the risk was higher in women. Nevertheless, when the sample was stratified by sex and age simultaneously, the risk was shown to be the highest among females aged 50–74 years, except for HTG, which was higher in older males. The lowest risk was shown among females aged 18–50, except for LHDL-C. These findings can be explained by the direct effect of menopause, which strongly influences changes in lipid metabolism (particularly HDL-C) with a lack of estrogens and progesterone, as well as the disturbed ratio of estrogens and progesterone to androgens and corticosteroids (64). There is an interplay between vitamin D and other steroids, including estrogens, androgens, and corticosteroids, and there is a similarity and synergy in the effects of vitamin D and estrogens in many metabolic pathways: vitamin D can potentiate the effect of estrogen and vice versa, and estrogen may increase 1,25(OH)D in postmenopausal women (30, 54, 65–69).

One of the reasons for the higher risk of LHDL-C among both post- and premenopausal women could be the usage of inadequate cut-offs for LHDL-C among the studied female population. The selected cut-offs for LHDL-C may not be appropriate for the Asian female populations. For example, in the Korean female population, which in general has lower average levels of HDL-C but without metabolic risk, and the cut-offs are set at lower values of HDL-C (70). Using the cut-offs of other populations may erroneously classify the majority of the studied female population as “dyslipidemic”, while in fact, they are just in the normal range. However, there can also be other explanations.

As we all know, the concentration of 25(OH)D can be enhanced by exposure to ultraviolet irradiation (71, 72), and the epidemiological studies have proven that ultraviolet irradiation has a positive impact on cardiovascular diseases, obesity and metabolic syndrome (33). We observed a dose-response relationship between vitamin D and lipid profiles across UV indices and seasons. At the same concentration of 25(OH)D, we found that lower UV indices and winter were associated with a higher prevalence of abnormal lipid profiles. The mechanism involves low endogenously synthesized vitamin D levels due to low UV exposure, which hinders the utilization of 25 (OH) D and increases the incidence of lipid profile abnormalities (72–75). Also lifestyle changes due to the cold season (physical inactivity, high calorie diet) contribute to the increased prevalence of dyslipidemia. More interestingly, we found that the rates of HDL-C and RC abnormalities were higher in summer + fall and at higher UV indices. Previous studies have found the association between vitamin D and lipid profiles to be seasonal. Vitamin D levels were strongly correlated with HDL-C and TC levels in the summer/fall, and there was a weak correlation with HDL-C in the winter/spring (76). Vitamin D showed a negative correlation with HDL-C and DHCR7 at higher UV indices (48, 77). In the skin, high UV-B induces reduction of the DHCR7 expression and decreases its substrate 7DHC, which leads to a decrease in cholesterol synthesis, and increases 1α,25(OH)_2_D_3_ production (45).

In this study, we conducted a comprehensive analysis using a large sample size from the general population and a specific threshold for 25(OH)D concentration in relation to lipid profile has been determined. Furthermore, the study investigated the nonlinear relationships between 25(OH)D and lipid profile across different ultraviolet index and seasons. However, the present study is subject to certain limitations. The cross-sectional nature of the study prevents us from drawing conclusions about the causal relationship between 25(OH)D and lipid profile, future analysis of follow-up cohort data is needed to further investigate causality. The RC concentration was not directly measured, which may deviate from the true level. Additionally, some parameters (e.g., parathyroid hormone, insulin), for which significant roles in the relationship between 25(OH)D and dyslipidmia are described, were not assessed. In addition, these participants came from a single center, with a limited sample size, and all were Asian. Therefore, the generalization of the results in different racial or ethnic groups may require further validation of the corresponding large samples. In addition, the optimal HDL-C cutoff to predict CVA-IHD was 43 mg/dL and 48 mg/dL for Korean men and women, respectively, and 41 mg/dL and 56 mg/dL for US men and women, respectively (70). We, therefore, question if the cut-offs used in general (Eurupoid population) are relevant for our population, which could have distinctive cut-offs for HDL-C (more similar as in the Korean population). Finally, we did not asses the concentration of 1,25(OH), which can have an opposite effect compared to 25(OH)D on cholesterol synthesis in the liver (47), and the imbalance between two metabolites can influence cholesterol synthesis in the liver and other tissues.

## 5 Conclusion

Nonlinear inverse U-shaped associations were observed between 25(OH)D levels and abnormal lipid profile. The risk was particularly increased among females aged 50-74 years, then males, during the winter/spring period. In vitamin D deficient subjects [25(OH)D < 20 ng/ml], a positive association of serum vitamin D levels with the risk for dyslipidemia was observed, which data is needed to further investigate causality.

## Data availability statement

The raw data supporting the conclusions of this article will be made available by the authors, without undue reservation.

## Ethics statement

The studies involving humans were approved by the Ethics Committee of the Second Hospital of Dalian Medical University (grant number: 2022064). The studies were conducted in accordance with the local legislation and institutional requirements. The human samples used in this study were acquired from primarily isolated as part of your previous study for which ethical approval was obtained. Written informed consent for participation was not required from the participants or the participants' legal guardians/next of kin in accordance with the national legislation and institutional requirements.

## Author contributions

QW: Data curation, Writing – original draft, Conceptualization, Methodology, Visualization. XM: Data curation, Formal analysis, Methodology, Writing – review & editing, Supervision, Conceptualization, Investigation, Validation. MH: Visualization, Writing – review & editing, Conceptualization, Methodology, Validation. FX: Software, Visualization, Writing – review & editing, Data curation. GT: Validation, Visualization, Writing – review & editing, Conceptualization, Methodology. YH: Writing – review & editing, Formal analysis, Methodology. ZS: Methodology, Writing – review & editing, Conceptualization, Software. WZ: Writing – review & editing, Investigation, Project administration, Validation. XN: Visualization, Writing – review & editing, Validation. SL: Conceptualization, Funding acquisition, Resources, Supervision, Writing – review & editing, Data curation.
