# Supplementary material for: Nonlinear relationship between serum 25-hydroxyvitamin D and lipid profile in Chinese adults
Source: Front Nutr. 2024 Jun 12;11:1388017. doi: 10.3389/fnut.2024.1388017 (PMC11199867; doi:10.3389/fnut.2024.1388017)
Supplement: Supplementary file 1 [file Data_Sheet_1.pdf]

Supplementary Material

1 Nonlinear relationship between serum 25-hydroxyvitamin D and lipid profile in Chinese adults

2 Supplementary Figures and Tables

2.1 Supplementary Figures

(1) Fig.S1 Population screening process in this study depicted through a flow chart

(2) Fig.S2 The distribution of 25-hydroxyvitamin D concentration in sex, age, season, and ultraviolet index

(3) Fig.S3 Dose-response relationship between 25-hydroxyvitamin D and the risk for specific dyslipidemias stratified by sex in the additional regression model 4

(4) Fig.S4 Dose-response relationship between 25-hydroxyvitamin D and the risk for specific dyslipidemias stratified by age in the additional regression model 4

(5) Fig.S5 Dose-response relationship between 25-hydroxyvitamin D and the risk for specific dyslipidemias stratified by sex+age in the additional regression model 4

(6) Fig.S6 Dose-response relationship between 25-hydroxyvitamin D and the risk for specific dyslipidemias stratified by season in the additional regression model 4

(7) Fig.S7 Dose-response relationship between 25-hydroxyvitamin D and the risk for specific dyslipidemias stratified by ultraviolet index in the additional regression model 4

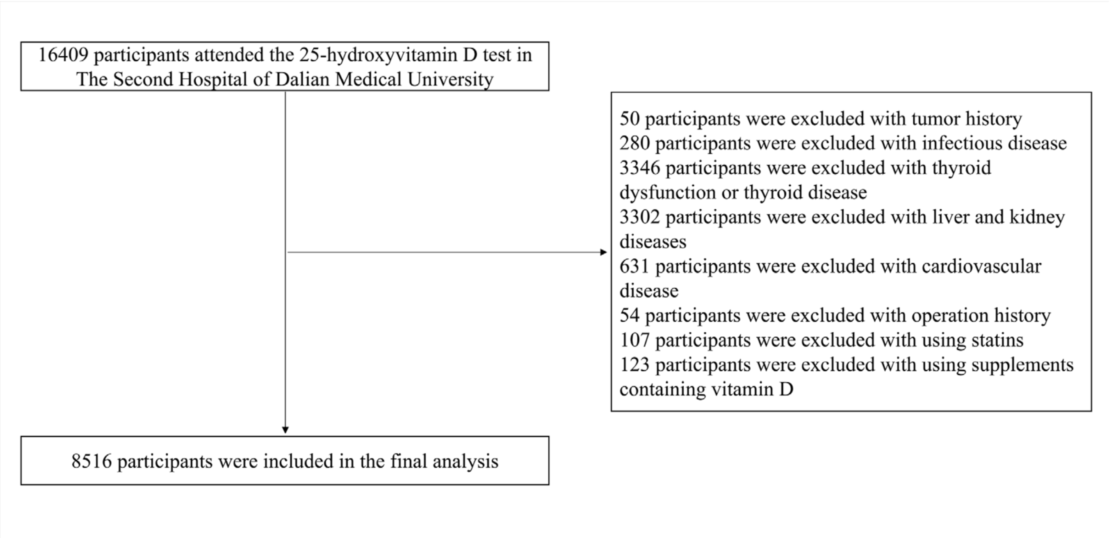

Fig.S1 Population screening process in this study depicted through a flow chart

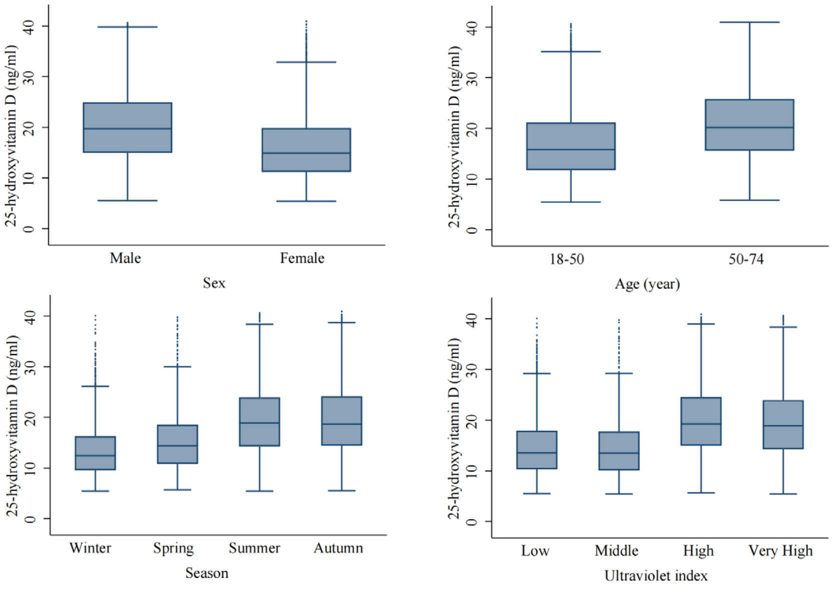

Fig.S2 The distribution of 25-hydroxyvitamin D concentration in sex, age, season, and ultraviolet index

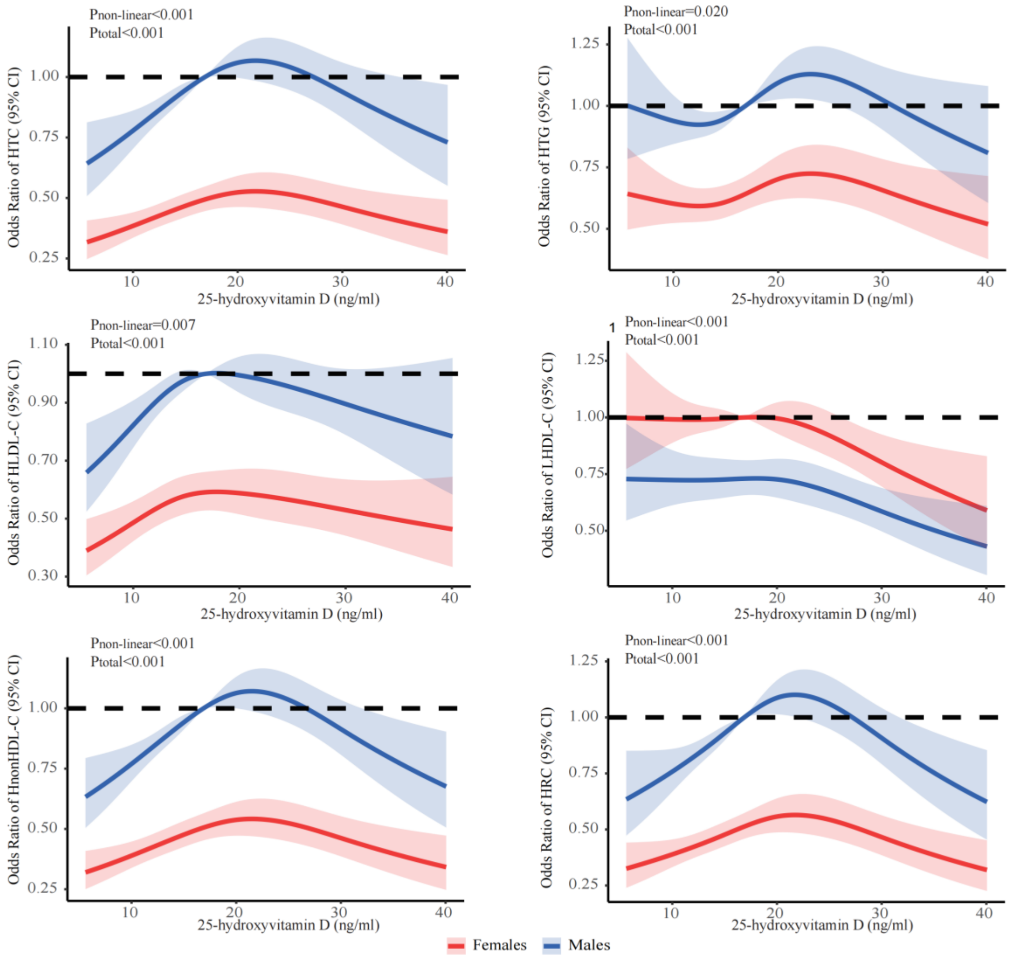

Fig.S3 Dose-response relationship between 25-hydroxyvitamin D and the risk for specific dyslipidemias stratified by sex in the additional regression model 4

Adjusted for covariates including sex (categorical), age (continuous), season (categorical), hypertension (categorical), diabetes (categorical), hyperuricemia (categorical), BMI (continuous), AC (continuous), ALT, AST, Globulin, γ-GGT, ALP, Tbil, Urea, and UA (all continuous)

CI: confidence interval, HTC: hypercholesterolemia, HTG: hypertriglyceridemia, LHDL-C: hypo-high-density lipoprotein cholesterolemia, HLDL-C: hyper-low-density lipoprotein cholesterol cholesterolemia, HnonHDL-C: hyper-non-high-density lipoprotein cholesterol, HRC: high remnant cholesterol

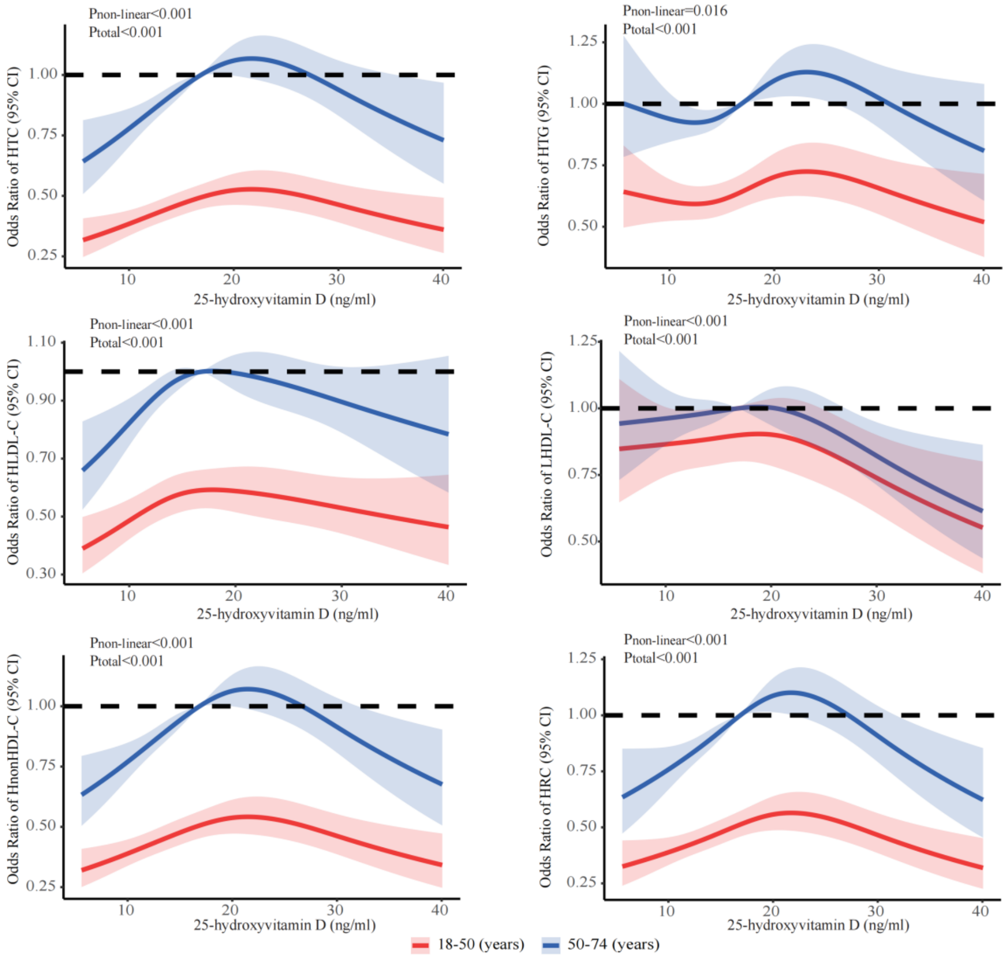

Fig.S4 Dose-response relationship between 25-hydroxyvitamin D and the risk for specific dyslipidemias stratified by age in the additional regression model 4

Adjusted for covariates including sex (categorical), age (continuous), season (categorical), hypertension (categorical), diabetes (categorical), hyperuricemia (categorical), BMI (continuous), AC (continuous), ALT, AST, Globulin, γ-GGT, ALP, Tbil, Urea, and UA (all continuous)

CI: confidence interval, HTC: hypercholesterolemia, HTG: hypertriglyceridemia, LHDL-C: hypo-high-density lipoprotein cholesterolemia, HLDL-C: hyper-low-density lipoprotein cholesterol cholesterolemia, HnonHDL-C: hyper-non-high-density lipoprotein cholesterol, HRC: high remnant cholesterol

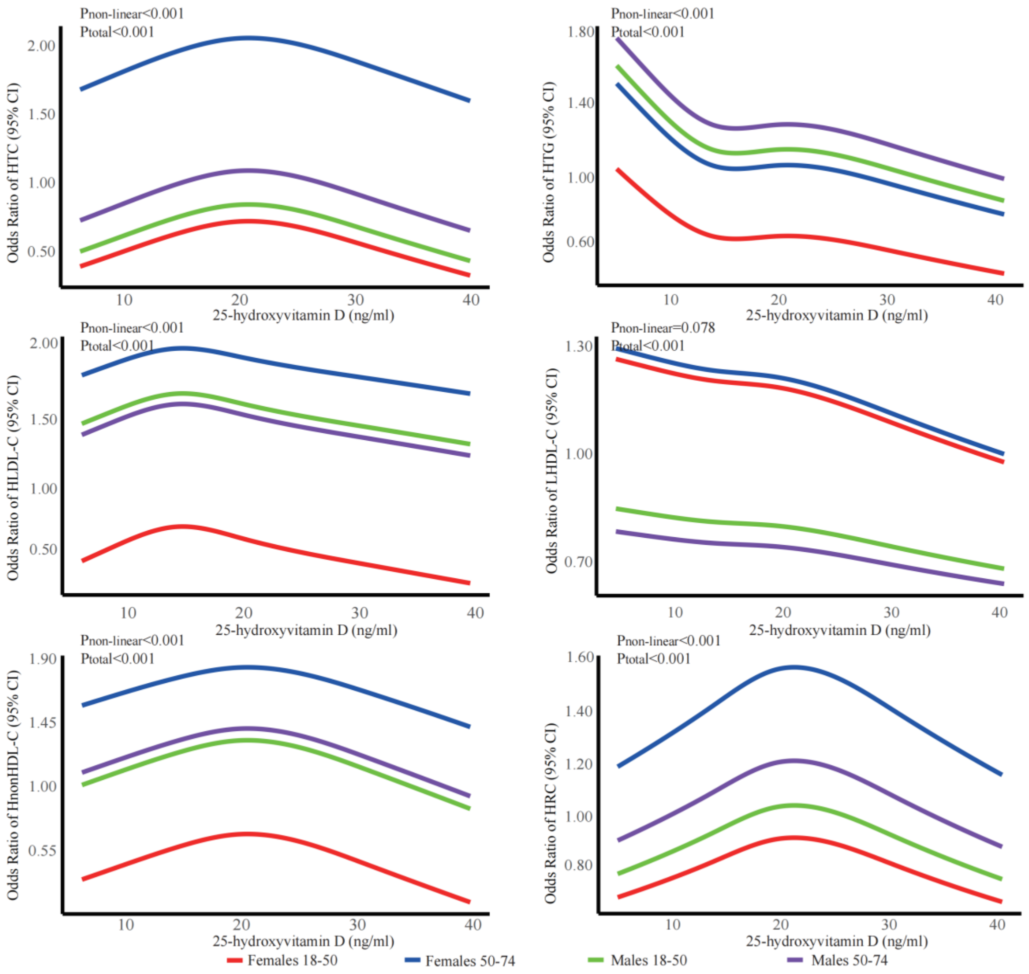

Fig.S5 Dose-response relationship between 25-hydroxyvitamin D and the risk for specific dyslipidemias stratified by sex+age in the additional regression model 4

Adjusted for covariates including sex (categorical), age (continuous), season (categorical), hypertension (categorical), diabetes (categorical), hyperuricemia (categorical), BMI (continuous), AC (continuous), ALT, AST, Globulin, γ-GGT, ALP, Tbil, Urea, and UA (all continuous)

CI: confidence interval, HTC: hypercholesterolemia, HTG: hypertriglyceridemia, LHDL-C: hypo-high-density lipoprotein cholesterolemia, HLDL-C: hyper-low-density lipoprotein cholesterol cholesterolemia, HnonHDL-C: hyper-non-high-density lipoprotein cholesterol, HRC: high remnant cholesterol

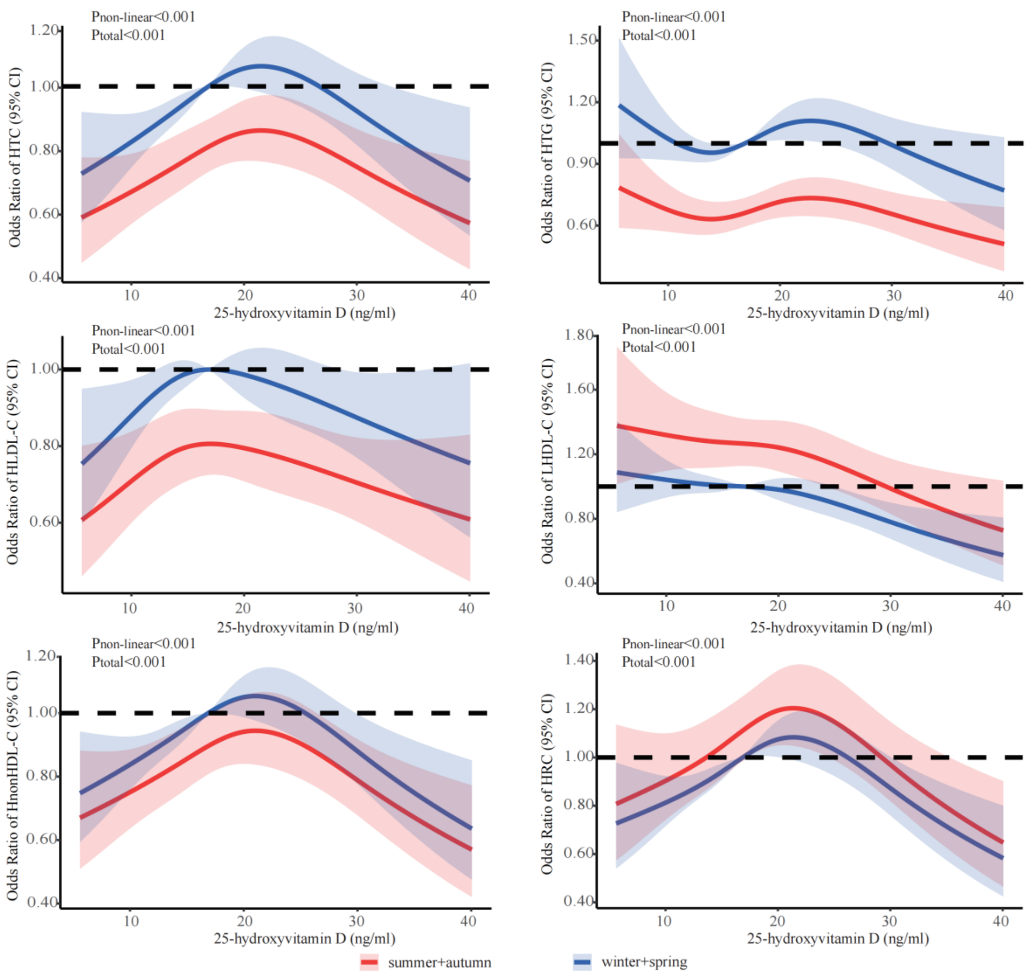

Fig.S6 Dose-response relationship between 25-hydroxyvitamin D and the risk for specific dyslipidemias stratified by season in the additional regression model 4

Adjusted for covariates including sex (categorical), age (continuous), season (categorical), hypertension (categorical), diabetes (categorical), hyperuricemia (categorical), BMI (continuous), AC (continuous), ALT, AST, Globulin, γ-GGT, ALP, Tbil, Urea, and UA (all continuous)

CI: confidence interval, HTC: hypercholesterolemia, HTG: hypertriglyceridemia, LHDL-C: hypo-high-density lipoprotein cholesterolemia, HLDL-C: hyper-low-density lipoprotein cholesterol cholesterolemia, HnonHDL-C: hyper-non-high-density lipoprotein cholesterol, HRC: high remnant cholesterol

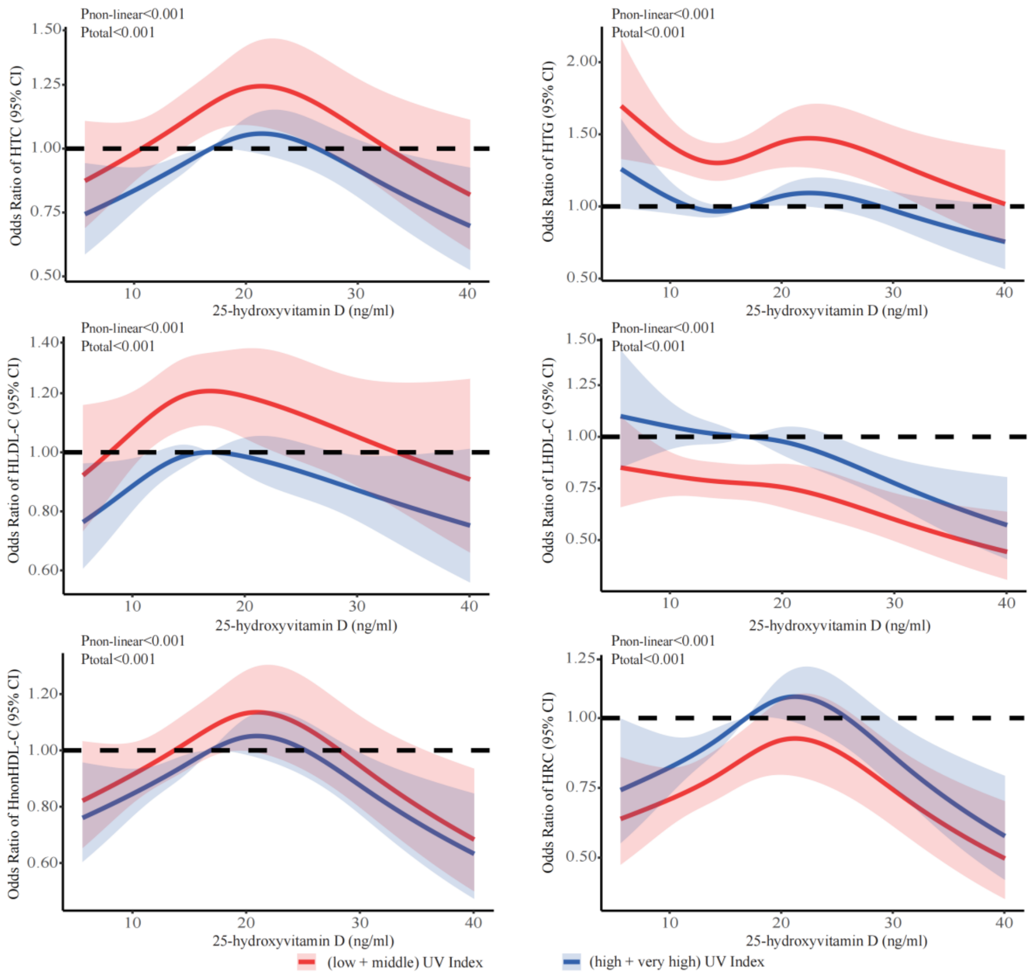

Fig.S7 Dose-response relationship between 25-hydroxyvitamin D and the risk for specific dyslipidemias stratified by ultraviolet index in the additional regression model 4

Adjusted for covariates including sex (categorical), age (continuous), season (categorical), hypertension (categorical), diabetes (categorical), hyperuricemia (categorical), BMI (continuous), AC (continuous), ALT, AST, Globulin, γ-GGT, ALP, Tbil, Urea, and UA (all continuous)

CI: confidence interval, HTC: hypercholesterolemia, HTG: hypertriglyceridemia, LHDL-C: hypo-high-density lipoprotein cholesterolemia, HLDL-C: hyper-low-density lipoprotein cholesterol cholesterolemia, HnonHDL-C: hyper-non-high-density lipoprotein cholesterol, HRC: high remnant cholesterol

2.2 Supplementary Tables

(1) Table S1 Post hoc analysis of variables with skewed distribution at different 25-hydroxyvitamin D concentrations

(2) Table S2 Post hoc analysis of variables with normal distribution at different 25-hydroxyvitamin D concentrations

(3) Table S3 Post hoc analysis of categorical variables at different 25-hydroxyvitamin D concentrations

(4) Table S4 The comparison of lipid levels between different 25-hydroxyvitamin D analyzed using analysis of covariance (ANCOVA) with adjustments for sex, age, and BMI

(5) Table S5 Post hoc analysis of ANCOVA for lipid concentrations at different 25-hydroxyvitamin D concentrations with adjustments for sex, age, and BMI

(6) Table S6 The VIFs (Variance Inflation Factors, as measures of the multicollinearity) in the additional regression model 4

(7) Table S7 The associations between 25(OH)D and lipid profile in the additional regression model 4

Table S1 Post hoc analysis of variables with skewed distribution at different 25-hydroxyvitamin D concentrations

Variables	>30 vs 20-30		>30 vs 10-20		>30 vs ≤10		20-30 vs 10-20		20-30 vs ≤10		10-20 vs ≤10	
	Std.Test Statistic	P Value	Std.Test Statistic	P Value	Std.Test Statistic	P Value	Std.Test Statistic	P Value	Std.Test Statistic	P Value	Std.Test Statistic	P Value
Age (years)	5.32	<0.001	12.46	<0.001	20.12	<0.001	12.24	<0.001	21.76	<0.001	14.43	<0.001
TG (mmol/L)	0.16	1.000	3.65	0.002	5.74	<0.001	6.80	<0.001	8.36	<0.001	3.99	<0.001
ALT (U/L)	0.82	1.000	6.55	<0.001	11.56	<0.001	10.10	<0.001	15.35	<0.001	9.08	<0.001
AST (U/L)	1.95	0.309	7.65	<0.001	10.50	<0.001	9.96	<0.001	12.42	<0.001	6.03	<0.001
GGT (U/L)	1.74	0.491	8.62	<0.001	13.89	<0.001	12.07	<0.001	17.48	<0.001	9.93	<0.001
ALP (U/L)	1.17	1.000	5.24	<0.001	7.65	<0.001	7.13	<0.001	9.36	<0.001	4.83	<0.001
Tbil (μmol/L)	0.91	1.000	3.11	0.011	5.78	<0.001	7.21	<0.001	9.36	<0.001	4.76	<0.001
Urea (mmol/L)	4.70	<0.001	10.48	<0.001	15.21	<0.001	9.89	<0.001	15.60	<0.001	9.52	<0.001

TG: Triglyceride, ALT: Alanine aminotransferase, AST: Aspartate aminotransferase, GGT: γ-glutamyl transferase, ALP: Alkaline phosphatase

Table S2 Post hoc analysis of variables with normal distribution at different 25-hydroxyvitamin D concentrations

Variables	>30 vs 20-30		>30 vs 10-20		>30 vs ≤10		20-30 vs 10-20		20-30 vs ≤10		10-20 vs ≤10	
	Mean Difference (95%CI)	P Value	Mean Difference (95%CI)	P Value	Mean Difference (95%CI)	P Value	Mean Difference (95%CI)	P Value	Mean Difference (95%CI)	P Value	Mean Difference (95%CI)	P Value
AC (cm)	0.42 (-0.83, 1.67)	1.000	3.70 (2.50, 4.89)	<0.001	7.45 (6.06, 8.84)	<0.001	3.28 (2.60, 3.95)	<0.001	7.03 (6.05, 8.01)	<0.001	3.75 (2.84, 4.66)	<0.001
BMI (kg/m^2)	-0.06 (-0.59, 0.48)	1.000	0.77 (0.26, 1.29)	<0.001	2.12 (1.52, 2.72)	<0.001	0.83 (0.54, 1.12)	<0.001	2.18 (1.76, 2.60)	<0.001	1.35 (0.95, 1.74)	<0.001
TC (mmol/L)	-0.02 (-0.12, -0.08)	<0.001	0.08 (-0.02, 0.17)	0.169	0.21 (0.10, 0.32)	<0.001	0.10 (0.04, 0.15)	<0.001	0.23 (0.15, 0.31)	<0.001	0.13 (0.06, 0.21)	<0.001
HDL-C (mmol/L)	0.01 (-0.03, 0.06)	0.326	-0.04 (-0.08, -0.01)	0.034	-0.11 (-0.15, -0.06)	<0.001	-0.06 (-0.08, -0.03)	<0.001	-0.11 (-0.15, -0.08)	<0.001	-0.07 (-0.09, -0.03)	<0.001
LDL-C (mmol/L)	-0.01 (-0.10, 0.07)	0.684	0.09 (0.01, 0.17)	0.330	0.21 (0.11, 0.30)	<0.001	0.10 (0.05, 0.15)	<0.001	0.22 (0.15, 0.29)	<0.001	0.12 (0.05, 0.18)	<0.001
nonHDL-C (mmol/L)	-0.03 (-0.14, 0.07)	0.325	0.12 (0.02, 0.22)	0.006	0.31 (0.20, 0.43)	<0.001	0.16 (0.10, 0.21)	<0.001	0.35 (0.27, 0.43)	<0.001	0.19 (0.12, 0.27)	<0.001
RC (mmol/L)	-0.02 (-0.6, 0.02)	0.936	0.04 (-0.01, 0.07)	0.056	0.11 (0.07, 0.15)	<0.001	0.55 (0.04, 0.08)	<0.001	0.13 (0.10, 0.16)	<0.001	0.07 (0.05, 0.10)	<0.001
Globulin (g/L)	-0.46 (-0.86, -0.05)	0.017	-0.62 (-1.00, -0.23)	<0.001	-0.62 (-1.07, -0.17)	0.002	-0.16 (-0.37, 0.06)	0.353	-0.16 (-0.48, 0.15)	1.000	-0.01 (-0.30, 0.29)	1.000

AC: Abdominal circumference, BMI: Body mass index, TC: Total cholesterol, HDL-C: High density lipoprotein cholesterol, LDL-C: Low Density Lipoprotein, nonHDL-C: non-high-density lipoprotein cholesterol, RC: Remnant cholesterol

Table S3 Post hoc analysis of categorical variables at different 25-hydroxyvitamin D concentrations

Variables	>30 vs 20-30	>30 vs 10-20	>30 vs ≤10	20-30 vs 10-20	20-30 vs ≤10	10-20 vs ≤10
	P Value	P Value	P Value	P Value	P Value	P Value
Sex	<0.001	<0.001	<0.001	<0.001	<0.001	<0.001
Age (years)	<0.001	<0.001	<0.001	<0.001	<0.001	<0.001
Season	0.015	<0.001	<0.001	<0.001	<0.001	<0.001
Ultraviolet index	0.034	<0.001	<0.001	<0.001	<0.001	<0.001
Hypertension	0.229	<0.001	<0.001	<0.001	<0.001	0.018
Diabetes	0.952	<0.001	<0.001	<0.001	<0.001	0.022
Hyperuricemia	0.887	<0.001	<0.001	<0.001	<0.001	<0.001

Table S4 The comparison of lipid levels between different 25-hydroxyvitamin D analyzed using analysis of covariance (ANCOVA) with adjustments for sex, age, and BMI

	Mean Square	F Value	P Value
HTC	0.673	2.9	0.033
HTG	1.144	5.4	0.001
HLDL-C	0.547	2.8	0.048
LHDL-C	0.575	6.2	0.013
HnonHDL-C	0.826	3.7	0.011
HRC	0.694	4.0	0.007

HTC: hypercholesterolemia, HTG: hypertriglyceridemia, LHDL-C: hypo-high-density lipoprotein cholesterolemia, HLDL-C: hyper-low-density lipoprotein cholesterolemia, HnonHDL-C: hyper-non-high-density lipoprotein cholesterol, HRC: high remnant cholesterol. Age, sex, and BMI were included as covariates in the model

Table S5 Post hoc analysis of ANCOVA for lipid concentrations at different 25-hydroxyvitamin D concentrations with adjustments for sex, age, and BMI

	>30 vs 20-30		>30 vs 10-20		>30 vs ≤10		20-30 vs 10-20		20-30 vs ≤10		10-20 vs ≤10	
	Mean Difference (95%CI)	P Value	Mean Difference (95%CI)	P Value	Mean Difference (95%CI)	P Value	Mean Difference (95%CI)	P Value	Mean Difference (95%CI)	P Value	Mean Difference (95%CI)	P Value
HTC	-0.05 (-0.11, 0.10)	0.169	0.04 (0.01, 0.10)	0.023	0.05 (0.01, 0.08)	<0.001	0.02 (0.01, 0.04)	<0.001	0.04 (0.01, 0.09)	<0.001	0.03 (0.01, 0.07)	<0.001
HTG	-0.0, (-0.09, 0.02)	0.760	-0.04 (-0.09, 0.02)	0.402	0.09 (0.03, 0.16)	0.002	-0.01 (-0.04, 0.03)	1.000	0.06 (0.01, 0.11)	0.006	0.05 (0.01, 0.10)	0.005
HLDL-C	-0.03 (-0.09, 0.03)	0.784	0.05 (0.02, 0.10)	0.010	0.25 (0.09, 0.43)	<0.001	0.04 (0.02, 0.15)	<0.001	0.03 (0.10, 0.56)	<0.001	0.27 (0.15, 0.49)	<0.001
LHDL-C	-0.03 (-0.08, 0.03)	1.000	-0.04 (-0.09, 0.01)	0.285	0.11 (0.05, 0.23)	<0.001	0.04 (0.01, 0.09)	<0.001	0.07 (0.02, 0.17)	<0.001	0.29 (0.01, 0.49)	<0.001
HnonHDL-C	-0.06 (-0.12, -0.01)	0.042	0.05 (0.01, 0.11)	0.001	0.08 (0.01, 0.14)	<0.001	0.02 (0.01, 0.42)	<0.001	0.04 (0.01, 0.11)	<0.001	0.03 (0.01, 0.07)	<0.001
HRC	-0.05 (-0.10, -0.01)	<0.001	0.03 (0.01, 0.08)	0.048	0.05 (0.01, 0.07)	0.007	0.03 (0.01, 0.05)	<0.001	0.04 (0.01, 0.09)	0.045	0.04 (0.01, 0.10)	<0.001

HTC: hypercholesterolemia, HTG: hypertriglyceridemia, LHDL-C: hypo-high-density lipoprotein cholesterolemia, HLDL-C: hyper-low-density lipoprotein cholesterolemia, HnonHDL-C: hyper-non-high-density lipoprotein cholesterol, HRC: high remnant cholesterol. Age, sex, and BMI were included as covariates in the model

Table S6 The VIFs (Variance Inflation Factors, as measures of the multicollinearity) in the additional regression model 4

Variables	Collinearity Statistics	
	Tolerance	VIFs
Sex	-	1.225
Age (year)	0.814	1.229
Season	-	1.282
AC (cm)	0.417	2.395
BMI (kg/m2)	0.601	1.664
ALT (U/L)	0.254	3.935
AST (U/L)	0.296	3.375
Globulin (g/L)	0.951	1.052
GGT (U/L)	0.691	1.448
ALP (U/L)	0.843	1.186
Tbil (μmol/L)	0.952	1.050
Urea (mmol/L)	0.876	1.142
UA (μmol/L)	0.621	1.609
Hypertension	-	1.098
Diabetes	-	1.061
Hyperuricemia	-	1.131

AC: Abdominal circumference, BMI: Body mass index, ALT: Alanine aminotransferase, AST: Aspartate aminotransferase, GGT: γ-glutamyl transferase, ALP: Alkaline phosphatase, UA: Urea acid

Table S7 The associations between 25(OH)D and lipid profile in the additional regression model 4

	Model 4	
	OR (95%CI)	P value
HTC		
25(OH)D suffciency	1	
25(OH)D insufficiency	1.524 (1.264, 1.837)	**<0.001**
25(OH)D deficiency	1.692 (1.463, 1.955)	**<0.001**
Severe 25(OH)D deficiency	1.395 (1.218, 1.597)	**<0.001**
HTG		
25(OH)D suffciency	1	
25(OH)D insufficiency	1.226 (1.013, 1.484)	**0.036**
25(OH)D deficiency	1.364 (1.178, 1.580)	**<0.001**
Severe 25(OH)D deficiency	1.092 (0.858, 1.391)	0.249
HLDL-C		
25(OH)D suffciency	1	
25(OH)D insufficiency	1.517 (1.250, 1.842)	**<0.001**
25(OH)D deficiency	1.701 (1.467, 1.971)	**<0.001**
Severe 25(OH)D deficiency	1.448 (1.265, 1.658)	**<0.001**
LHDL-C		
25(OH)D suffciency	1	
25(OH)D insufficiency	1.223 (0.978, 1.530)	0.078
25(OH)D deficiency	1.322 (1.067, 1.638)	**0.011**
Severe 25(OH)D deficiency	1.304 (1.019, 1.667)	**0.035**
HnonHDL		
25(OH)D suffciency	1	
25(OH)D insufficiency	1.776 (1.475, 2.138)	**<0.001**
25(OH)D deficiency	2.161 (1.874, 2.492)	**<0.001**
Severe 25(OH)D deficiency	1.559 (1.369, 1.776)	**<0.001**
HRC		
25(OH)D suffciency	1	
25(OH)D insufficiency	1.796 (1.450, 2.225)	**<0.001**
25(OH)D deficiency	2.182 (1.841, 2.587)	**<0.001**
Severe 25(OH)D deficiency	1.564 (1.329, 1.839)	**<0.001**

Model 4 was adjusted for covariates including sex (categorical), age (continuous), season (categorical), hypertension (categorical), diabetes (categorical), hyperuricemia (categorical), BMI (continuous), AC (continuous), ALT, AST, Globulin, GGT, ALP, Tbil, Urea, and UA (all continuous)

25(OH)D: 25-hydroxyvitamin D, OR: odds ratio, CI: confidence interval. HTC: hypercholesterolemia, HTG: hypertriglyceridemia, LHDL-C: hypo-high-density lipoprotein cholesterolemia, HLDL-C: hyper-low-density lipoprotein cholesterol cholesterolemia, HnonHDL: hyper-non-high-density lipoprotein cholesterol, HRC: high remnant cholesterol

Bold font indicates statistically significant differences (P<0.05)
